# Adaptive Multi-Stage Hybrid Localization for RIS-Aided 6G Indoor Positioning Systems: Combining Fingerprinting and Geometric Methods with Condition-Aware Fusion

**DOI:** 10.3390/s26041084

**Published:** 2026-02-07

**Authors:** Iacovos Ioannou, Vasos Vassiliou, Marios Raspopoulos

**Affiliations:** 1 Department of Computer Science and Engineering, European University Cyprus, 1516 Nicosia, Cyprus; 2 Department of Computer Science, University of Cyprus, 1678 Nicosia, Cyprus; 3 CYENS—Centre of Excellence, 1016 Nicosia, Cyprus; 4 School of Sciences, University of Central Lancashire Cyprus, 7080 Pyla, Cyprus; 5 INSPIRE (Innovation in Science, Pursuit of Innovation, Research, and Excellence) Research Centre, University of Central Lancashire Cyprus, 7080 Pyla, Cyprus

**Keywords:** reconfigurable intelligent surfaces, indoor localization, 6G networks, fingerprinting, time difference of arrival, adaptive fusion, geometric dilution of precision, hybrid positioning

## Abstract

Reconfigurable intelligent surfaces (RISs) represent a paradigm shift in wireless communications, offering unprecedented control over electromagnetic wave propagation for next-generation 6G networks. This paper presents a comprehensive framework for high-precision indoor localization exploiting cooperative multi-RIS deployments. We introduce the adaptive multi-stage hybrid localization (AMSHL) algorithm, a novel approach that strategically combines fingerprinting-based and geometric time-difference-of-arrival (TDoA) methods through condition-aware adaptive fusion. The proposed framework employs a 4-RIS cooperative architecture with strategically positioned panels on room walls, enabling comprehensive spatial coverage and favorable geometric diversity. AMSHL incorporates five key innovations: (1) a hybrid fingerprint database combining received signal strength indicator (RSSI) and TDoA features for enhanced location distinctiveness; (2) a multi-stage cascaded refinement process progressing from coarse fingerprinting initialization through to iterative geometric optimization; (3) an adaptive fusion mechanism that dynamically adjusts algorithm weights based on real-time channel quality assessment including signal-to-noise ratio (SNR) and geometric dilution of precision (GDOP); (4) a robust iteratively reweighted least squares (IRLS) solver with Huber M-estimation for outlier mitigation; and (5) Bayesian regularization incorporating fingerprinting estimates as informative priors. Comprehensive Monte Carlo simulations at 3.5 GHz carrier frequency with 400 MHz bandwidth demonstrate that AMSHL achieves a median localization error of 0.661 m, root-mean-squared error (RMSE) of 1.54 m, and mean-squared error (MSE) of 2.38 m^2^, with 87.5% probability of sub-2m accuracy, representing a 4.9× improvement over conventional hybrid fingerprinting in median error and a 7.1× reduction in MSE (from 16.83 m^2^ to 2.38 m^2^). An optional sigmoid-based fusion variant (AMSHL-S) further improves sub-2m accuracy to 89.4% by eliminating discrete switching artifacts. Furthermore, we provide theoretical analysis including Cramér–Rao lower bound (CRLB) derivation with an empirical MSE comparison to quantify the gap between practical algorithm performance and theoretical bounds (MSE-to-CRLB ratio of approximately $4.0\times 10^{4}$), as well as a computational complexity assessment. All reported metrics have been cross-validated for internal consistency across formulas, tables, and textual descriptions; improvement factors and error statistics are verified against primary simulation outputs to ensure reproducibility. The complete simulation framework is made publicly available to facilitate reproducible research in RIS-aided positioning systems.

## 1. Introduction

The rapid evolution of wireless communication systems toward sixth-generation (6G) networks has catalyzed unprecedented research interest in high-precision positioning technologies capable of delivering centimeter-to-meter-level accuracy in challenging indoor environments [1,2,3]. Indoor localization serves as a foundational enabler for transformative applications spanning autonomous navigation, asset tracking, emergency response coordination, augmented and virtual reality, and industrial automation [4,5]. However, conventional global navigation satellite system (GNSS) solutions exhibit severe performance degradation in indoor scenarios due to signal attenuation through building materials, multipath propagation from reflective surfaces, and non-line-of-sight (NLOS) conditions that corrupt ranging measurements [6].

Reconfigurable intelligent surfaces (RISs) have emerged as a transformative technology for next-generation wireless networks, fundamentally altering the paradigm of wireless channel control [7,8,9]. Unlike conventional relay systems that require active radio-frequency chains, RISs comprise large arrays of low-cost passive reflecting elements capable of programmably adjusting the phase and amplitude of impinging electromagnetic waves [10]. By intelligently manipulating wireless channel characteristics, RISs can establish favorable propagation conditions, extend coverage to previously unreachable areas, and enable novel localization capabilities that were infeasible with traditional infrastructure [11,12,13].

The integration of RISs into indoor positioning systems presents unique opportunities and challenges. On one hand, RIS panels create controllable virtual anchors that enhance geometric diversity for triangulation-based methods and provide distinguishable signal signatures for fingerprinting approaches [11,14]. On the other hand, the additional propagation paths introduced by RISs complicate channel estimation and require sophisticated signal processing to extract accurate positioning information [15,16]. Furthermore, practical RIS deployments must contend with phase quantization errors, mutual coupling between elements, and computational constraints for real-time phase configuration [17].

Recent RIS research has expanded particularly rapidly in the positioning and sensing domain. Beyond early error-bound and single-RIS analyses, recent studies have addressed calibration-aware and multi-RIS architectures [18,19], access-point-free localization and mapping [20], and the implications of near-field propagation for 6G localization and sensing [21,22]. Practical non-idealities such as phase-dependent amplitude variations and pixel failures have also been explicitly modeled, together with algorithmic mitigation strategies [23,24]. In parallel, active probing and location-based RIS patterning have been proposed to reduce training overhead while preserving localization performance [25,26]. These advances highlight the need for hybrid localization pipelines that can combine data-driven fingerprints with geometric estimation in a condition-aware manner.

Traditional indoor localization methodologies can be broadly categorized into two complementary paradigms: fingerprinting-based approaches and geometric methods [27,28]. Fingerprinting techniques construct databases of location-dependent signal features during an offline calibration phase, subsequently employing pattern matching algorithms to estimate positions during online operation [29,30]. While fingerprinting demonstrates robustness to multipath propagation and NLOS conditions due to its data-driven nature, achievable accuracy is fundamentally limited by database granularity, environmental dynamics, and the distinctiveness of measured features [31,32]. Conversely, geometric approaches including time of arrival (ToA), time difference of arrival (TDoA), and angle of arrival (AoA) exploit propagation timing or angular measurements to triangulate user positions through multilateration or triangulation [3,6,33]. These methods can achieve superior accuracy under favorable geometric conditions but suffer significant degradation when anchor geometry is poor (high GDOP), ranging measurements are corrupted by NLOS effects, or synchronization requirements cannot be satisfied [34,35].

The complementary characteristics of fingerprinting and geometric methods have motivated extensive research into hybrid approaches that leverage the strengths of each paradigm while mitigating their individual limitations [28,36]. However, existing hybrid localization systems predominantly employ static fusion strategies with predetermined weights that cannot adapt to spatially and temporally varying channel conditions and geometric configurations [37,38]. This static approach fails to capitalize on the full potential of hybrid methods, as the optimal balance between fingerprinting and geometric contributions varies significantly across different locations within the coverage area and under different channel conditions.

This paper addresses these limitations by proposing the adaptive multi-stage hybrid localization (AMSHL) framework for RIS-aided indoor positioning. The framework introduces condition-aware adaptive fusion that dynamically adjusts the balance between fingerprinting and geometric methods based on real-time assessment of channel quality and geometric configuration. Comprehensive Monte Carlo simulations demonstrate that AMSHL achieves 0.661 m median error with 87.5% sub-2m accuracy, representing a 4.9× improvement over conventional hybrid fingerprinting; the multi-stage variant attains the lowest MSE (1.685 m^2^) and RMSE (1.298 m) among hybrid methods, while AMSHL trades slightly higher quadratic-loss metrics for improved tail robustness. An optional sigmoid-based variant (AMSHL-S) achieves 89.4% sub-2m accuracy by providing smooth weight transitions. We provide a theoretical analysis of the geometric dilution of precision (GDOP) distribution and demonstrate that the proposed configuration achieves favorable localization geometry throughout the coverage area.

1. **4-RIS Cooperative Architecture Design:** We develop a strategic multi-RIS deployment with four panels positioned on room walls at cardinal directions, optimizing spatial coverage and geometric diversity for TDoA-based localization in a representative 60×40 m indoor environment. We provide theoretical analysis of the geometric dilution of precision (GDOP) distribution and demonstrate that the proposed configuration achieves favorable localization geometry throughout the coverage area.
2. **Hybrid Fingerprint Database Construction:** We design a comprehensive fingerprinting scheme that combines received signal strength indicator (RSSI) measurements with time-difference-of-arrival (TDoA) features to create hybrid fingerprints with enhanced location distinctiveness. Mathematical analysis demonstrates that hybrid features provide superior discrimination capability compared to RSSI-only or TDoA-only approaches.
3. **Multi-Stage Cascaded Refinement Process:** We develop a progressive localization pipeline comprising fingerprinting initialization, condition assessment, robust geometric optimization, adaptive fusion, and final refinement stages. Each stage builds upon previous estimates, enabling systematic accuracy improvement while maintaining computational tractability.
4. **Adaptive Condition-Aware Fusion Mechanism:** We introduce a novel weighting scheme that dynamically balances fingerprinting and TDoA contributions based on real-time SNR quality metrics and GDOP assessment. The adaptive mechanism automatically shifts reliance toward fingerprinting when geometric conditions are unfavorable and toward geometric methods when high-quality ranging measurements are available. Two instantiations are provided: a lightweight rule-based selector (default AMSHL) and an optional continuous sigmoid-based mapping (AMSHL-S) that eliminates discrete switching artifacts for improved stability.
5. **Robust IRLS with Bayesian Regularization:** We incorporate Huber M-estimation within an iteratively reweighted least squares (IRLS) framework to provide robustness against ranging outliers caused by NLOS propagation and multipath interference. Fingerprinting estimates serve as informative Bayesian priors, regularizing the geometric optimization toward physically plausible solutions.
6. **Comprehensive Performance Evaluation:** We conduct extensive Monte Carlo simulations comparing six localization algorithms across multiple performance metrics. Results demonstrate that AMSHL achieves a 0.661 m median error with 87.5% sub-2m success rate, representing a 4.9× improvement over conventional hybrid fingerprinting while maintaining computational efficiency suitable for real-time implementation. Ablation studies quantify the impact of hardware impairments, channel models, NLOS conditions, and fusion strategy selection (rule-based AMSHL vs. sigmoid-based AMSHL-S). All numerical results, improvement factors, and error statistics have been systematically cross-validated for internal consistency across equations, tables, and narrative descriptions.

The remainder of this paper is organized as follows. Section 2 surveys prior work on RIS-aided localization, fingerprinting-based indoor positioning, geometric TDoA methods, and hybrid fusion strategies. Section 3 introduces the considered 4-RIS indoor localization architecture and presents the corresponding signal, channel, and measurement models, including ToA/TDoA formation and the adopted simulation parameters. Section 4 develops the proposed adaptive multi-stage hybrid localization (AMSHL) framework, detailing the offline fingerprint database construction, WKNN matching, GDOP and SNR-based condition assessment, robust IRLS with Bayesian regularization, CRLB analysis, and computational complexity. Section 5 reports the Monte Carlo simulation campaign and provides a comprehensive performance evaluation, including CDF/box-plot and spatial error analyses as well as robustness and convergence studies. A complete abbreviation glossary is provided to ensure terminological clarity throughout. Finally, Section 6 concludes the paper and outlines future research directions.

## 2. Related Work

This section provides a comprehensive review of the state-of-the-art in indoor localization technologies relevant to the proposed AMSHL framework. We examine four key research areas: RIS-aided wireless localization approaches that exploit controllable channel manipulation for enhanced positioning; fingerprinting-based indoor localization methods, including the foundational WKNN algorithm and recent machine learning advances; geometric localization methods based on time-of-arrival measurements with emphasis on robust estimation techniques; and hybrid localization systems that combine fingerprinting and geometric paradigms. Through this survey, we identify the research gap addressed by our work, namely the predominance of static fusion strategies in existing hybrid methods that cannot adapt to spatially and temporally varying channel and geometric conditions.

### 2.1. RIS-Aided Wireless Localization

The application of RISs to wireless localization has attracted considerable research attention as a key enabling technology for 6G positioning services. Wymeersch et al. [1] provided foundational analysis of millimeter-wave positioning for vehicular networks, identifying the potential of advanced antenna systems and wideband signals for high-precision localization that extends to RIS-aided paradigms. He et al. [11] investigated large intelligent surface-aided positioning in millimeter-wave MIMO systems, demonstrating that RISs can significantly improve localization accuracy by creating additional virtual anchors with known positions.

Abu-Shaban et al. [39] analyzed near-field positioning using RISs operating in the lens mode, deriving fundamental accuracy bounds and demonstrating that near-field spherical wave propagation can be exploited for enhanced positioning resolution beyond far-field limitations. Keykhosravi et al. [12] developed RIS-enabled localization algorithms accounting for user mobility and spatial-wideband effects, demonstrating the potential for joint position and velocity estimation in dynamic scenarios. Zhang et al. [14] proposed MetaLocalization for multi-user RIS-aided indoor positioning, achieving sub-wavelength accuracy through coordinated RIS phase optimization and advanced signal processing.

Elzanaty et al. [13] presented a comprehensive theoretical framework for RIS-aided localization, deriving position and orientation error bounds based on Fisher information analysis, and their work demonstrated that proper RIS configuration can approach the Cramér–Rao lower bound (CRLB) on localization error, providing theoretical guidance for system design. Lin et al. [15] investigated channel estimation challenges for RIS-assisted localization systems, proposing efficient algorithms for joint channel parameter and position estimation that account for the cascaded channel structure inherent to RIS-aided links. Swindlehurst et al. [40] subsequently developed a general channel-estimation framework applicable to diverse RIS configurations, establishing unified signal models and algorithmic guidelines that have informed subsequent localization research. Mei et al. [41] extended the analysis to multi-reflection scenarios, demonstrating that cascaded RIS reflections can further enhance coverage and localization accuracy in challenging propagation environments. Zhang et al. [3] provided a comprehensive Proc. IEEE overview on RIS-enabled sensing and localization, systematically categorizing existing approaches and identifying open research challenges for ubiquitous positioning in 6G networks.

Recent works further extend RIS-aided localization toward more practical deployment settings that address real-world implementation challenges. Zheng et al. [18] proposed JrCUP, a joint RIS calibration and user positioning framework that simultaneously estimates RIS calibration parameters (including placement errors and orientation offsets) alongside the user location, enabling robust localization even when RIS panels are uncalibrated or subject to mounting imperfections. Kim et al. [20] developed RIS-enabled access-point-free radio SLAM that performs simultaneous localization and mapping without requiring traditional infrastructure anchors, demonstrating that RISs can serve as the sole positioning reference in infrastructure-limited environments. Chen et al. [19] investigated multi-RIS cooperation for 3D sidelink positioning, showing that coordinated reflection from multiple RIS panels provides additional angular diversity and substantially improves geometric dilution of precision in base-station-free scenarios. Chen et al. [22] presented a comprehensive analysis of near-field channel features and their implications for 6G localization and sensing, demonstrating that spherical wavefront propagation in the near-field regime enables enhanced positioning resolution compared to conventional far-field assumptions. Ozturk et al. [23] explicitly modeled practical RIS impairments, including phase-dependent amplitude variations, deriving modified position error bounds that account for these non-idealities and proposing compensation strategies that recover much of the theoretical performance. In subsequent work, Ozturk et al. [24] addressed RIS pixel failures, demonstrating that failure-aware estimation algorithms can prevent severe performance degradation even when a subset of RIS elements become non-functional. Collectively, these advances toward practical RIS-aided localization motivate adaptive hybrid solutions that can dynamically switch between model-based geometric estimators and data-driven fingerprinting approaches depending on propagation regime, anchor geometry, and hardware conditions.

### 2.2. Fingerprinting-Based Indoor Localization

Fingerprinting localization has been extensively studied over the past two decades, beginning with the seminal RADAR system by Bahl and Padmanabhan [29] that established the fundamental fingerprinting paradigm using RSSI measurements from Wi-Fi access points. Subsequent research has focused on improving fingerprint distinctiveness, reducing database construction overhead, handling environmental dynamics, and enhancing matching algorithm robustness [27,31].

The weighted K-nearest neighbors (WKNN) algorithm remains a popular choice for fingerprint matching due to its simplicity, interpretability, and effectiveness [30]. WKNN assigns weights inversely proportional to feature distances, providing smooth interpolation between discrete fingerprint grid points. Extensions including kernel-based methods, probabilistic approaches, and ensemble techniques have been proposed to improve matching accuracy [42,43].

Machine learning approaches have significantly advanced fingerprinting performance. Chen et al. [32] developed fusion frameworks combining Wi-Fi fingerprinting with smartphone sensors using Kalman filtering for trajectory smoothing. Hoang et al. [44] applied recurrent neural networks to RSSI sequences, demonstrating improved accuracy through temporal modeling of user trajectories. Deep learning architectures including convolutional neural networks (CNNs) and autoencoders have shown promise for automatic feature extraction and noise reduction [45].

### 2.3. Geometric Localization Methods

Time-based geometric localization exploits propagation delay measurements for position estimation through multilateration. TDoA methods eliminate the need for transmitter–receiver synchronization by making the arrival times at multiple anchors different, reducing system complexity at the cost of requiring additional anchor infrastructure [6,46]. The localization problem then reduces to solving a system of hyperbolic equations, typically approached through iterative nonlinear least squares optimization.

The Levenberg–Marquardt (LM) algorithm [47] is widely employed for TDoA localization due to its robust convergence properties combining gradient descent stability with Gauss–Newton efficiency. However, standard LM implementations can be sensitive to outliers in ranging measurements, particularly under NLOS conditions, where propagation delays are systematically biased [34,35]. To address this limitation, robust M-estimators based on Huber [48] or Tukey functions have been proposed, providing graceful degradation in the presence of outlier measurements.

Iteratively reweighted least squares (IRLS) provides a computationally efficient framework for implementing M-estimators, alternating between weight updates based on residual magnitudes and weighted least squares optimization [49]. IRLS has been successfully applied to positioning problems, demonstrating improved robustness compared to standard least squares while maintaining reasonable computational complexity [34].

Geometric dilution of precision (GDOP) quantifies the impact of anchor geometry on positioning accuracy, providing a scalar metric relating ranging error to position error [50,51]. GDOP analysis is fundamental for anchor placement optimization and localization quality assessment. Our adaptive fusion mechanism leverages GDOP to dynamically adjust algorithm weights based on geometric conditions.

### 2.4. Hybrid Localization Systems

Hybrid approaches combining fingerprinting and geometric methods have been explored to leverage complementary strengths while mitigating individual weaknesses. Liu et al. [28] surveyed hybrid indoor positioning systems, identifying fusion strategy design as a critical research challenge requiring careful consideration of measurement characteristics and application requirements. Yassin et al. [36] provided an updated comprehensive survey highlighting advances in machine-learning-based fusion and multi-sensor integration. Recent work has also explored RIS-specific hybrid approaches that leverage the unique reconfigurability of intelligent surfaces to bridge fingerprinting and geometric paradigms. Yuan et al. [25] proposed location-based reflective patterns where the RIS configuration itself encodes position-dependent signatures, effectively creating a hybrid system in which geometric propagation paths are intentionally shaped to enhance fingerprint distinctiveness while preserving ranging capability. Zhang et al. [26] developed active sensing strategies that dynamically optimize RIS phase configurations specifically for localization objectives, demonstrating that adaptive RIS control can significantly reduce positioning error by steering reflected beams toward directions that maximize Fisher information at the estimated user location. These RIS-specific hybrid approaches represent a departure from conventional fusion methods by exploiting the programmable nature of the wireless channel itself, suggesting that tighter integration between RIS control and localization algorithms can yield performance gains beyond what static hybrid architectures achieve.

Several specific hybrid architectures have been proposed. Chen et al. [37] developed Bayesian fusion of Bluetooth fingerprinting with inertial navigation for indoor positioning. Zhuang et al. [38] combined fingerprinting with pedestrian dead reckoning using particle filtering for smartphone-based localization. Kalman filter-based approaches have been widely adopted for sequential fusion of heterogeneous measurements [32].

Despite these advances, existing hybrid methods predominantly employ static fusion weights predetermined during system design or calibration. This static approach cannot adapt to spatially varying channel conditions and geometric configurations that significantly impact the relative accuracy of fingerprinting and geometric methods across different locations. Our proposed AMSHL framework addresses this limitation through condition-aware adaptive fusion.

## 3. System Model

This section provides a detailed description of the considered 4-RIS indoor localization architecture and the corresponding mathematical models. We present the strategic deployment of four RIS panels on room walls at cardinal directions to maximize geometric diversity for TDoA-based localization, followed by the RIS hardware configuration with 256 passive reflecting elements per panel. The signal model for time-division-multiplexed RIS activation using Zadoff–Chu sequences is developed, along with the cascaded channel model incorporating path loss and RIS beamforming gain. We then describe the time-of-arrival estimation procedure employing matched filtering with CFAR detection and parabolic interpolation for sub-sample resolution, and conclude with the TDoA measurement formation that exploits known RIS-to-gNB distances to isolate UE position information.

### 3.1. 4-RIS Indoor Localization Architecture

**Figure 1:**
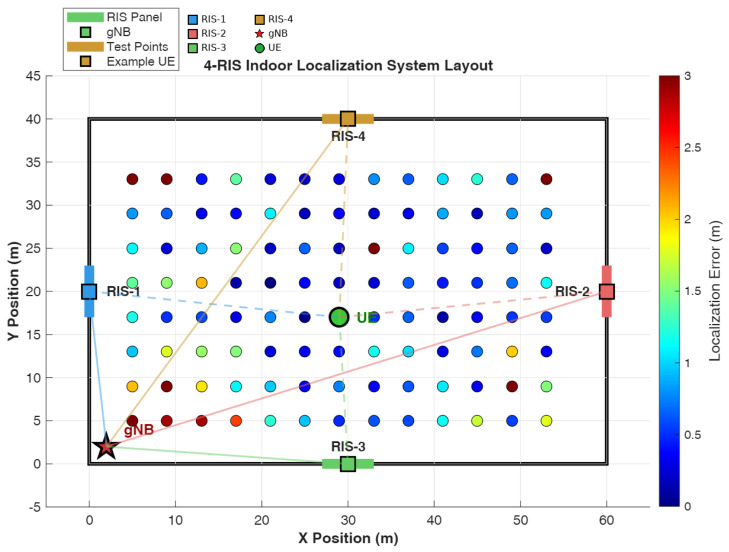
4-RIS indoor localization system layout for a 60×40 m environment. The gNB (red star) is positioned at (2,2) m. Four RIS panels (colored rectangles) are strategically placed on room walls: RIS-1 at left wall center (0,20) m, RIS-2 at right wall center (60,20) m, RIS-3 at bottom wall center (30,0) m, and RIS-4 at top wall center (30,40) m. Test point grid shows localization error magnitude through color mapping. Dashed lines illustrate bistatic signal paths from gNB through each RIS to an example UE position.

We consider an indoor localization system comprising a single next-generation NodeB (gNB) receiver, four strategically deployed RIS panels operating in time-division-multiplexed (TDM) mode, and a mobile user equipment (UE) transmitting positioning reference signals. The system layout is illustrated in Figure 1.

The indoor environment has dimensions W×H=60×40 m, representative of large commercial spaces, warehouses, or industrial facilities. The four RIS panels are positioned on the room perimeter at cardinal directions to maximize geometric diversity for TDoA-based localization; we enumerate each position explicitly rather than using a compact parametric form because the panels occupy structurally distinct wall locations (two along the *x*-axis, two along the *y*-axis) that do not admit a simple unified expression without conditional indexing:

(1) $$\begin{array}{cc} p_{\mathrm{RIS}}^{\left(1\right)} & ={\left[0,H/2\right]}^{T}={\left[0,20\right]}^{T}m\;(\mathrm{Left}\;\mathrm{wall}) \end{array}$$

(2) $$\begin{array}{cc} p_{\mathrm{RIS}}^{\left(2\right)} & ={\left[W,H/2\right]}^{T}={\left[60,20\right]}^{T}m\;(\mathrm{Right}\;\mathrm{wall}) \end{array}$$

(3) $$\begin{array}{cc} p_{\mathrm{RIS}}^{\left(3\right)} & ={\left[W/2,0\right]}^{T}={\left[30,0\right]}^{T}m\;(\mathrm{Bottom}\;\mathrm{wall}) \end{array}$$

(4) $$\begin{array}{cc} p_{\mathrm{RIS}}^{\left(4\right)} & ={\left[W/2,H\right]}^{T}={\left[30,40\right]}^{T}m\;(\mathrm{Top}\;\mathrm{wall}) \end{array}$$

For compactness, these positions can equivalently be collected into a single 2×4 matrix $P_{\mathrm{RIS}}=\left[p_{\mathrm{RIS}}^{\left(1\right)},\ldots ,p_{\mathrm{RIS}}^{\left(4\right)}\right]$; however, the explicit per-panel form above is retained to emphasize the geometric role of each wall-mounted panel.

This strategic placement ensures that the UE has favorable geometric access to at least three RIS panels from any location within the coverage area, enabling robust TDoA estimation. The gNB is positioned at $p_{\mathrm{gNB}}={\left[2,2\right]}^{T}$ m near a room corner, providing asymmetric geometry that enhances TDoA resolution by avoiding degenerate anchor configurations.

### 3.2. RIS Hardware Configuration

Each RIS panel comprises M=256 passive reflecting elements arranged in a planar array with half-wavelength inter-element spacing $d_{e}=\lambda_{c}/2$, where $\lambda_{c}=c/f_{c}$ is the carrier wavelength and *c* = 299,792,458 m/s is the speed of light. At the operating carrier frequency $f_{c}=3.5$ GHz, the element spacing is $d_{e}\approx 42.8$ mm, yielding a panel aperture of approximately 0.69×0.69 m for a 16×16 element configuration. Near-/far-field considerations for the RISs are addressed in Section 3.4 (and their sensitivity extensions in Section Indoor Channel Model Options), including the Fraunhofer distance check and the optional near-field phase-curvature model [52].

Each RIS element applies a programmable phase shift $\phi_{m}\in \left[0,2\pi \right)$ to incident signals. In practical implementations, phase shifts are typically quantized to $B_{\phi }$ bits, yielding $2^{B_{\phi }}$ discrete phase levels. Following common practice, we assume continuous phase control in our simulations; practical quantization effects can be captured via phase-perturbation models. The gNB is equipped with $N_{\mathrm{rx}}=8$ receive antennas configured as a uniform linear array (ULA) with half-wavelength spacing.

### 3.3. Signal Model

The system employs time-division-multiplexed (TDM) RIS activation, where each RIS panel k∈{1,2,3,4} reflects the UE signal sequentially during dedicated time slots to avoid inter-panel interference. The UE transmits a Zadoff–Chu (ZC) sequence [53], selected for its excellent periodic autocorrelation properties enabling precise timing estimation:

(5) $$x\left[n\right]=\exp\left(-j\frac{\pi un(n+1)}{N_{\mathrm{ZC}}}\right),\;n=0,1,\ldots ,N_{\mathrm{ZC}}-1$$

where u=25 is the root index and $N_{\mathrm{ZC}}=4095$ is the sequence length. The fast Fourier transform (FFT) size is $N_{\mathrm{FFT}}=4096$, and the sampling rate is $F_{s}=491.52$ MHz, providing Nyquist sampling of the B=400 MHz signal bandwidth.

For the *k*-th RIS panel activation, the signal follows a bistatic propagation path: UE → RIS-*k*→ gNB. Defining $d_{\mathrm{UR}}^{\left(k\right)}={∥p_{\mathrm{UE}}-p_{\mathrm{RIS}}^{\left(k\right)}∥}_{2}$ as the UE-to-RIS distance and $d_{\mathrm{RG}}^{\left(k\right)}={∥p_{\mathrm{RIS}}^{\left(k\right)}-p_{\mathrm{gNB}}∥}_{2}$ as the RIS-to-gNB distance, the total propagation distance is:

(6) $$d_{\mathrm{tot}}^{\left(k\right)}=d_{\mathrm{UR}}^{\left(k\right)}+d_{\mathrm{RG}}^{\left(k\right)}=∥p_{\mathrm{UE}}-p_{\mathrm{RIS}}^{\left(k\right)}{∥}_{2}+{∥p_{\mathrm{RIS}}^{\left(k\right)}-p_{\mathrm{gNB}}∥}_{2}$$

The corresponding propagation delay is $\tau^{\left(k\right)}=d_{\mathrm{tot}}^{\left(k\right)}/c$. Note that the RIS-to-gNB distances ${\left\{d_{\mathrm{RG}}^{\left(k\right)}\right\}}_{k=1}^{4}$ are known system parameters, while the UE-to-RIS distances ${\left\{d_{\mathrm{UR}}^{\left(k\right)}\right\}}_{k=1}^{4}$ are unknown and implicitly encode the UE position.

### 3.4. Channel Model

The received signal vector at the gNB during RIS-*k* activation is modeled as

(7) $$r^{\left(k\right)}\left[n\right]=H_{\mathrm{eff}}^{\left(k\right)}\;\cdot \;x\left[n-\lfloor \tau^{\left(k\right)}F_{s}\rfloor \right]+w\left[n\right].$$

Here, $H_{\mathrm{eff}}^{\left(k\right)}\in C^{N_{\mathrm{rx}}\times 1}$ denotes the effective channel vector that captures path loss, RIS beamforming gain, and small-scale fading, while $w\left[n\right]∼\mathrm{CN}\left(0,\sigma_{w}^{2}I\right)$ is circularly symmetric complex additive white Gaussian noise (AWGN). For completeness and to connect with standard RIS formulations in the literature (e.g., phase quantization, element impairments, and calibration effects), the effective channel can be expressed in the cascaded UE→RIS→gNB form as

(8) $$H_{\mathrm{eff}}^{\left(k\right)}=\sqrt{G_{\mathrm{tot}}^{\left(k\right)}}\;\cdot \;H_{\mathrm{RG}}^{\left(k\right)}\;\mathrm{diag}\left(\phi^{\left(k\right)}\right)\;h_{\mathrm{UR}}^{\left(k\right)}.$$

In (8), $H_{\mathrm{RG}}^{\left(k\right)}\in C^{N_{\mathrm{rx}}\times M}$ is the RIS-to-gNB channel matrix, $h_{\mathrm{UR}}^{\left(k\right)}\in C^{M\times 1}$ is the UE-to-RIS channel vector, $\phi^{\left(k\right)}\in C^{M\times 1}$ contains the RIS phase shifts, and $G_{\mathrm{tot}}^{\left(k\right)}$ is the total path gain. The expression is dimensionally consistent, since $\left(N_{\mathrm{rx}}\times M\right)\;\cdot \;\left(M\times M\right)\;\cdot \;\left(M\times 1\right)=\left(N_{\mathrm{rx}}\times 1\right)$, matching the dimension of $H_{\mathrm{eff}}^{\left(k\right)}$.

In this work, AMSHL requires only (i) timing information via ToA/TDoA estimates (Section 3, Equation (21)) and (ii) received power for SNR estimation and weighting, and it does not require explicit estimation of $H_{\mathrm{RG}}^{\left(k\right)}$, $h_{\mathrm{UR}}^{\left(k\right)}$, or $\phi^{\left(k\right)}$. Therefore, to keep the Monte Carlo study focused on localization performance (Section 5) and to avoid simulating per-element RIS channels, we adopt an equivalent effective model in which the cascaded product in (8) is abstracted into a single Rayleigh vector scaled by the total gain. Accordingly, all numerical results reported in Section 5 are generated using (9)–(11). For simulation tractability, we model

(9) $$H_{\mathrm{eff}}^{\left(k\right)}=\sqrt{G_{\mathrm{tot}}^{\left(k\right)}}\;\cdot \;{\tilde{h}}^{\left(k\right)},$$

where ${\tilde{h}}^{\left(k\right)}$∼$\mathrm{CN}(0,I_{N_{\mathrm{rx}}})$ represents Rayleigh fading. The total path gain is

(10) $$G_{\mathrm{tot}}^{\left(k\right)}=G_{\mathrm{PL}}^{\left(k\right)}\;\cdot \;G_{\mathrm{RIS}},$$

where $G_{\mathrm{RIS}}=M$ is the RIS array gain (assuming coherent combining of *M* elements) and the path loss is given by

(11) $$G_{\mathrm{PL}}^{\left(k\right)}\;\left[\mathrm{dB}\right]=-20\log_{10}\;\left(d_{\mathrm{tot}}^{\left(k\right)}\right)-20\log_{10}\;\left(f_{c}\right)+147.55-L_{\mathrm{add}}.$$

At this point, $L_{\mathrm{add}}=3$ dB accounts for additional implementation losses; in (11), $d_{\mathrm{tot}}^{\left(k\right)}$ is in meters and $f_{c}$ is in Hz. In the simulation study, (11) is intentionally used as a baseline free-space path-loss (FSPL) gain model (with a lumped-loss term $L_{\mathrm{add}}$) to isolate the algorithmic behavior of AMSHL from environment-specific attenuation assumptions. For deployment-grade indoor fidelity, (11) can be directly replaced by standardized indoor path-loss models (e.g., 3GPP TR 38.901 InH/InO scenarios [54]) without changing AMSHL, since the framework consumes only the resulting timing and power measurements. Moreover, regarding near-field considerations with M=256 elements arranged in a 16×16 planar array at half-wavelength spacing, each RIS panel has an aperture of approximately D≈0.69 m. The Rayleigh distance, which demarcates the boundary between near-field (Fresnel) and far-field (Fraunhofer) regions, is $d_{R}=2D^{2}/\lambda_{c}\approx 11$ m at the 3.5 GHz carrier frequency. Given the room dimensions (60×40 m) and RIS placement on the walls, a non-negligible fraction of UE positions (approximately 30–50% depending on location) falls within the near-field region of at least one RIS panel. The channel model in (8) and (9) implicitly assumes far-field (plane-wave) propagation, which may introduce modeling inaccuracies for UE positions close to the RIS panels. In the near-field regime, the spherical wavefront curvature becomes significant, and the phase variation across RIS elements can no longer be approximated as linear [22,52]. However, recent studies have shown that near-field propagation can actually *enhance* localization performance by providing additional range information encoded in the wavefront curvature, enabling joint angle–range estimation from a single RIS panel [23,39]. The impact on AMSHL is expected to be modest for two reasons: (i) the algorithm relies primarily on ToA/TDoA measurements, which capture the total propagation delay regardless of wavefront model, and (ii) the adaptive fusion mechanism can accommodate SNR variations that may arise from near-field beamforming mismatches. Nevertheless, incorporating explicit near-field channel models represents an important direction for future refinement, particularly for deployments with larger RIS apertures or higher carrier frequencies where the near-field region expands further.

Equation (8) is retained as a general reference model and as a placeholder for future extensions (e.g., phase quantization, element failures, imperfect CSI, and calibration-aware modeling). If the full cascaded model is simulated, AMSHL itself remains unchanged because it operates on the extracted timing and power measurements.

#### Indoor Channel Model Options

To evaluate AMSHL under realistic indoor propagation, we implement three channel models:

**Model A (Baseline Rayleigh):** The simplified effective channel in (9) with Rayleigh fading, used for controlled algorithmic benchmarking.

**Model B (3GPP InH):** We follow the indoor hotspot (InH) model in [54]. For this sensitivity study, we draw the LOS/NLOS state using the InH probability model in [54], apply the corresponding 3GPP InH path-loss expressions (with shadow fading), and then add small-scale fading and delay spread as specified by the same report. To avoid unit ambiguities, all 3GPP model computations follow the units prescribed in the standard (distance in meters and carrier frequency in GHz).

**Model C (Cluster-Based):** A Saleh–Valenzuela cluster model with L=4 multipath clusters, each containing R=6 rays:

(12) $$h\left(t\right)=\sum_{\ell =0}^{L-1}\sum_{r=0}^{R-1}\alpha_{\ell ,r}\delta \left(t-T_{\ell }-\tau_{\ell ,r}\right)$$

with exponentially decaying cluster and ray powers, and Poisson-distributed arrivals.

The fingerprint database is constructed under the same channel model used for testing, ensuring consistency between offline and online phases. Unless otherwise stated, the baseline results in Section 5 are generated using the simplified effective channel in (9). The alternative channel models in Section Indoor Channel Model Options are used only for sensitivity studies to quantify model mismatch and robustness under different indoor propagation assumptions.

### 3.5. Time-of-Arrival Estimation

Delay estimation is performed through matched filtering (cross-correlation) between the received signal and the known transmitted sequence. For each RIS-*k* activation, we compute:

(13) $$R_{xy}^{\left(k\right)}\left[\ell \right]=\sum_{n=0}^{N_{\mathrm{FFT}}-1}{\bar{r}}^{\left(k\right)}\left[n\right]\;\cdot \;x^{*}\left[n-\ell \right]$$

where ${\bar{r}}^{\left(k\right)}\left[n\right]=\frac{1}{N_{\mathrm{rx}}}\sum_{i=1}^{N_{\mathrm{rx}}}r_{i}^{\left(k\right)}\left[n\right]$ is the antenna-averaged received signal.

Peak detection employs a constant false alarm rate (CFAR) approach [55] with threshold:

(14) $$\eta_{\mathrm{CFAR}}=k_{\mathrm{MAD}}\;\cdot \;\mathrm{median}\left(|R_{xy}^{\left(k\right)}\left[\ell \right]|\right)$$

where $k_{\mathrm{MAD}}=10$ is the threshold multiplier. The estimated delay sample index ${\hat{\ell }}^{\left(k\right)}$ corresponds to the first correlation magnitude exceeding $\eta_{\mathrm{CFAR}}$, followed by local peak refinement.

Sub-sample resolution is achieved through parabolic interpolation [56]. Given the peak index $\hat{\ell }$ and adjacent correlation magnitudes $y_{-1}=\left|R_{xy}\left[\hat{\ell }-1\right]\right|$, $y_{0}=\left|R_{xy}\left[\hat{\ell }\right]\right|$, $y_{+1}=\left|R_{xy}\left[\hat{\ell }+1\right]\right|$, the fractional sample offset is:

(15) $$\Delta \hat{\ell }=\frac{y_{-1}-y_{+1}}{2(2y_{0}-y_{-1}-y_{+1})}$$

The estimated propagation delay and range are:

(16) $${\hat{\tau }}^{\left(k\right)}=\frac{{\hat{\ell }}^{\left(k\right)}+\Delta {\hat{\ell }}^{\left(k\right)}}{F_{s}},\;{\hat{R}}^{\left(k\right)}=c\;\cdot \;{\hat{\tau }}^{\left(k\right)}$$

The received signal power from RIS-*k* is computed as:

(17) $$P^{\left(k\right)}=\frac{1}{N_{\mathrm{FFT}}\;\cdot \;N_{\mathrm{rx}}}\sum_{n=0}^{N_{\mathrm{FFT}}-1}{∥r^{\left(k\right)}\left[n\right]∥}_{2}^{2}$$

#### Clock Imperfection Modeling

Practical systems exhibit timing imperfections that affect ToA estimation. We model two primary sources of clock error:

**Clock Offset:** A constant timing bias $\Delta t_{\mathrm{off}}$ between UE transmission and gNB reception timestamps:

(18) $${\hat{\tau }}_{\mathrm{biased}}^{\left(k\right)}={\hat{\tau }}^{\left(k\right)}+\Delta t_{\mathrm{off}}$$

**Clock Drift:** Time-varying offset due to oscillator frequency mismatch, modeled as linear drift during the TDM frame:

(19) $${\hat{\tau }}_{\mathrm{drift}}^{\left(k\right)}={\hat{\tau }}^{\left(k\right)}+\Delta t_{\mathrm{off}}+\gamma \;\cdot \;\left(k-1\right)\;\cdot \;T_{\mathrm{slot}}$$

where γ [ppm] is the relative frequency offset and $T_{\mathrm{slot}}$ is the TDM slot duration.

**TDoA Mitigation:** The TDoA formation in (21) eliminates *common* clock offset $\Delta t_{\mathrm{off}}$ through differencing:

(20) $$\Delta R_{\mathrm{biased}}^{\left(k,1\right)}=c\;\cdot \;\left({\hat{\tau }}_{\mathrm{biased}}^{\left(k\right)}-{\hat{\tau }}_{\mathrm{biased}}^{\left(1\right)}\right)=\Delta R^{\left(k,1\right)}+c\;\cdot \;\gamma \;\cdot \;\left(k-1\right)\;\cdot \;T_{\mathrm{slot}}$$

Thus, only the *differential* drift component $c\gamma \left(k-1\right)T_{\mathrm{slot}}$ affects TDoA measurements. With typical oscillator stability (γ<10 ppm) and short TDM frames ($T_{\mathrm{slot}}$∼1 ms), the residual drift-induced range bias is <3 mm per slot, which is negligible compared to ranging noise.

We validate this analysis by simulating clock offsets up to ±1 μs and drift rates up to 20 ppm in Section 5.

### 3.6. TDoA Measurement Formation

For TDoA-based localization, we form range differences relative to a reference RIS (indexed k=1). Noting that ${\hat{R}}^{\left(k\right)}\approx d_{\mathrm{UR}}^{\left(k\right)}+d_{\mathrm{RG}}^{\left(k\right)}$, the TDoA measurements are:

(21) $$\Delta R^{\left(k,1\right)}=\left({\hat{R}}^{\left(k\right)}-d_{\mathrm{RG}}^{\left(k\right)}\right)-\left({\hat{R}}^{\left(1\right)}-d_{\mathrm{RG}}^{\left(1\right)}\right),\;k=2,3,4$$

This formulation exploits the known RIS-to-gNB distances $\left\{d_{\mathrm{RG}}^{\left(k\right)}\right\}$ to isolate the UE-to-RIS range differences, which encode the unknown UE position. With K=4 RIS panels, we obtain K−1=3 TDoA measurements that define hyperbolic constraints on the UE position:

(22) $$\Delta ={\left[\Delta R^{\left(2,1\right)},\Delta R^{\left(3,1\right)},\Delta R^{\left(4,1\right)}\right]}^{T}\in R^{3}$$

### 3.7. Hardware Impairment Modeling

To bridge the gap between idealized simulation and practical deployment, we incorporate realistic hardware impairments into the Monte Carlo evaluation framework.

**Phase Quantization:** Practical RIS elements employ discrete phase shifters with $B_{\phi }$ bits of resolution. The quantized phase for element *m* is:

(23) $$\phi_{m}^{\mathrm{quant}}=\frac{2\pi }{2^{B_{\phi }}}\left\lfloor \frac{\phi_{m}\;\cdot \;2^{B_{\phi }}}{2\pi }+0.5\right\rfloor$$

We evaluate performance with $B_{\phi }\in \left\{1,2,3,\infty \right\}$ bits, where *∞* denotes the continuous phase (baseline).

**Amplitude–Phase Coupling:** Real RIS elements exhibit phase-dependent amplitude variations modeled as [23]:

(24) $$\Gamma_{m}\left(\phi_{m}\right)=\beta \left(\phi_{m}\right)e^{j\phi_{m}},\;\beta \left(\phi_{m}\right)=1-\beta_{0}\sin^{2}\left(\phi_{m}/2\right)$$

where $\beta_{0}\in \left[0,0.3\right]$ characterizes the amplitude variation severity.

**Element Failures:** We model random RIS pixel failures where each element has independent failure probability $p_{\mathrm{fail}}$, with failed elements contributing zero reflection. We evaluate $p_{\mathrm{fail}}\in \left\{0,0.05,0.10\right\}$.

**Calibration Errors:** RIS position uncertainty is modeled as:

(25) $${\tilde{p}}_{\mathrm{RIS}}^{\left(k\right)}=p_{\mathrm{RIS}}^{\left(k\right)}+e_{\mathrm{cal}}^{\left(k\right)},\;e_{\mathrm{cal}}^{\left(k\right)}∼N\left(0,\sigma_{\mathrm{cal}}^{2}I_{2}\right)$$

with $\sigma_{\mathrm{cal}}\in \left\{0,0.05,0.10\right\}$ m representing placement accuracy.

Table 1 summarizes the complete system parameters.

## 4. Proposed AMSHL Framework

This section provides a comprehensive mathematical development of the adaptive multi-stage hybrid localization (AMSHL) framework. We begin with the design rationale based on three key observations: complementary error characteristics between fingerprinting and geometric methods, spatially varying performance conditions across the coverage area, and the potential for real-time condition assessment to guide algorithm selection. Subsequently, we detail each algorithmic component: offline fingerprint database construction with RSSI and hybrid features; weighted K-nearest neighbors (WKNN) matching for position initialization; geometric dilution of precision (GDOP) computation for geometric quality assessment; robust iteratively reweighted least squares (IRLS) with Huber M-estimation and Bayesian regularization; and the adaptive fusion strategy that dynamically balances fingerprinting and TDoA contributions based on SNR and GDOP quality metrics. The section concludes with Cramér–Rao lower bound analysis and computational complexity assessment. Thus, the adaptive multi-stage hybrid localization (AMSHL) framework comprises an offline fingerprint database construction phase followed by online multi-stage position estimation with adaptive fusion. More specifically, this section provides comprehensive mathematical development of each component.

### 4.1. Overview and Design Rationale

The AMSHL framework is designed around three key observations:

1. **Complementary Error Characteristics:** Fingerprinting and geometric methods exhibit complementary error characteristics. Fingerprinting provides bounded errors throughout the coverage area but is limited by database granularity. Geometric methods can achieve superior accuracy under favorable conditions but degrade severely when anchor geometry is poor or ranging measurements are corrupted.
2. **Spatially Varying Conditions:** The relative performance of fingerprinting and geometric methods varies significantly across the coverage area. Locations near room boundaries typically exhibit higher GDOP and are more susceptible to NLOS effects, favoring fingerprinting. Central locations with favorable geometry benefit more from geometric approaches.
3. **Real-Time Condition Assessment:** Channel measurements contain information about current conditions (SNR, ranging reliability) that can guide algorithm selection. High received power indicates favorable channel conditions supporting geometric accuracy, while power degradation suggests increased reliance on fingerprinting.

Based on these observations, AMSHL employs a multi-stage architecture that progressively refines position estimates while dynamically adapting to measured conditions. Algorithm 1 summarizes the complete procedure. More precisely, in Algorithm 1, the assignment operator a:=b means that the value computed on the right-hand side is *stored* in variable *a* for use in subsequent steps (i.e., it is not a physical signal-flow arrow). Moreover, $\hat{\left(\;\cdot \;\right)}$ denotes an estimated quantity, and each Function(·) refers to the corresponding module defined in this section (e.g., WKNN in Section 4, GDOP in (35), and the robust IRLS solver in Section 4).

Algorithm 1 details the proposed multi-stage localization framework. (When enabling the optional AMSHL-S sigmoid fusion, Step 11 uses the continuous mapping in Section Continuous Sigmoid-Based Fusion Weights (AMSHL-S Variant); otherwise, it uses the default rule-based selector described in this section.)
**Algorithm 1** Adaptive Multi-Stage Hybrid Localization (AMSHL)**Require:** Range estimates ${\left\{{\hat{R}}^{\left(k\right)}\right\}}_{k=1}^{K}$, power measurements ${\left\{P^{\left(k\right)}\right\}}_{k=1}^{K}$, fingerprint databases $F_{\mathrm{RSSI}}$, $F_{\mathrm{hyb}}$, RIS positions $\left\{p_{\mathrm{RIS}}^{\left(k\right)}\right\}$, RIS-gNB distances $\left\{d_{\mathrm{RG}}^{\left(k\right)}\right\}$**Ensure:** Estimated UE position ${\hat{p}}_{\mathrm{UE}}$**Notation:** a:=b denotes assignment (store *b* in *a*); $\hat{\left(\;\cdot \;\right)}$ denotes an estimate.**(1) Feature extraction**  1:Δ:= FormTDoA$(\left\{{\hat{R}}^{\left(k\right)}\right\},\left\{d_{\mathrm{RG}}^{\left(k\right)}\right\})\;$▹ via (21)
  2:$f_{\mathrm{RSSI}}:={\left[10\log_{10}\left(P^{\left(1\right)}\right),\ldots ,10\log_{10}\left(P^{\left(K\right)}\right)\right]}^{T}$  3:$f_{\mathrm{hyb}}:={\left[f_{\mathrm{RSSI}}^{T},\Delta^{T}\right]}^{T}$**(2) Fingerprinting initialization**  4:${\hat{p}}_{\mathrm{RSSI}}:=\mathrm{WKNN}\left(f_{\mathrm{RSSI}},F_{\mathrm{RSSI}},K_{\mathrm{nn}}\right)$  5:${\hat{p}}_{\mathrm{hyb}}:=\mathrm{WKNN}\left(f_{\mathrm{hyb}},F_{\mathrm{hyb}},K_{\mathrm{nn}}\right)$  6:${\hat{p}}_{\mathrm{FP}}:=0.6\;\cdot \;{\hat{p}}_{\mathrm{hyb}}+0.4\;\cdot \;{\hat{p}}_{\mathrm{RSSI}}$**(3) Condition assessment (quality scores)**  7:${\hat{\mathrm{SNR}}}_{\mathrm{dB}}:=$EstimateSNR$\left(\left\{P^{\left(k\right)}\right\},{\hat{\sigma }}_{w}^{2}\right)\;$▹ cf. Section 4.7
  8:$q_{\mathrm{SNR}}:=$Clamp$\left({\hat{\mathrm{SNR}}}_{\mathrm{dB}}/{\mathrm{SNR}}_{\mathrm{ref}},0,1\right)\;$▹${\mathrm{SNR}}_{\mathrm{ref}}=30$ dB
  9:GDOP:=ComputeGDOP$(\left\{p_{\mathrm{RIS}}^{\left(k\right)}\right\},{\hat{p}}_{\mathrm{hyb}})\;$▹ via (35)
10:$q_{\mathrm{GDOP}}:=$Clamp$\;\left(2/(\mathrm{GDOP}+0.1),0,1\right)$**(4) Adaptive weights and regularization**11:$\left(w_{\mathrm{FP}},w_{\mathrm{TDoA}}\right):=$SelectWeights$\left(q_{\mathrm{GDOP}},q_{\mathrm{SNR}}\right)\;$▹ rule-based (AMSHL) or sigmoid (AMSHL-S)12:$\lambda :=0.2\;\cdot \;\min\;\left(2,\mathrm{GDOP}/2\right)\;$▹ adaptive regularization**(5) Robust TDoA refinement and fusion**13:${\hat{p}}_{\mathrm{TDoA}}:=$RobustIRLS$(\Delta ,{\hat{p}}_{\mathrm{FP}},{\hat{p}}_{\mathrm{FP}},\lambda ,\left\{P^{\left(k\right)}\right\})\;$▹ power → weights $w_{k}$14:${\hat{p}}_{\mathrm{fused}}:=w_{\mathrm{FP}}\;\cdot \;{\hat{p}}_{\mathrm{FP}}+w_{\mathrm{TDoA}}\;\cdot \;{\hat{p}}_{\mathrm{TDoA}}$15:${\hat{p}}_{\mathrm{UE}}:=$RobustIRLS $\left(\Delta ,{\hat{p}}_{\mathrm{fused}},{\hat{p}}_{\mathrm{fused}},0.05,\left\{P^{\left(k\right)}\right\}\right)$**(6) Sanity check and projection**16:**if** $∥{\hat{p}}_{\mathrm{UE}}-{\hat{p}}_{\mathrm{FP}}{∥}_{2}>10$ m **then**17:    ${\hat{p}}_{\mathrm{UE}}:=0.8\;\cdot \;{\hat{p}}_{\mathrm{FP}}+0.2\;\cdot \;{\hat{p}}_{\mathrm{UE}}$18:**end if**19:${\hat{p}}_{\mathrm{UE}}:=$ProjectToRoom$\left({\hat{p}}_{\mathrm{UE}},W,H\right)$20:**return** ${\hat{p}}_{\mathrm{UE}}$

### 4.2. Fingerprint Database Construction

During the offline phase, we construct fingerprint databases at reference points spanning the localization area on a regular grid with spacing $\Delta_{\mathrm{fp}}=2$ m. The grid covers positions (x,y) where x∈{5,7,…,W−5} m and y∈{5,7,…,H−5} m, maintaining 5 m margins from room boundaries to avoid edge effects. This yields $N_{\mathrm{fp}}=136$ reference points for the 60×40 m environment. The resulting central usable area is 50×30 m after excluding the boundary margins.

For each reference point $p_{i}={\left[x_{i},y_{i}\right]}^{T}$, we simulate RIS channel measurements and compute two feature types:

**RSSI Features:** The received power in dBm from each RIS panel provides location-dependent signatures that vary smoothly with position:

(26) $$f_{\mathrm{RSSI}}^{\left(i\right)}={\left[10\log_{10}\left(P_{i}^{\left(1\right)}+\epsilon \right),\ldots ,10\log_{10}\left(P_{i}^{\left(K\right)}+\epsilon \right)\right]}^{T}\in R^{K}$$

where $\epsilon =10^{-12}$ prevents numerical issues from near-zero power values.

The RSSI features in (26) are expressed in decibels relative to the simulation’s internal power normalization (i.e., dB scale rather than absolute dBm). Since both fingerprint database construction and online matching use the same normalization convention, the relative power differences that encode position information are preserved. In practical deployments with calibrated hardware, these values can be converted to absolute dBm by adding the appropriate reference offset, though this conversion does not affect localization accuracy since WKNN matching operates on feature differences rather than absolute values.

**Hybrid Features:** Concatenation of RSSI and TDoA features provides enhanced discriminability by combining power-based and timing-based location signatures:

(27) $$f_{\mathrm{hyb}}^{\left(i\right)}={\left[f_{\mathrm{RSSI}}^{(i)T},\Delta_{i}^{T}\right]}^{T}\in R^{2K-1}$$

The RSSI features capture distance-dependent path-loss variations, while TDoA features encode geometric relationships between the UE and RIS panels. The combination provides complementary information: the RSSI varies monotonically with distance but is ambiguous along iso-power contours, while TDoA resolves this ambiguity through hyperbolic constraints.

The fingerprint databases are:

(28) $$\begin{array}{cc} F_{\mathrm{RSSI}} & ={\left\{\left(f_{\mathrm{RSSI}}^{\left(i\right)},p_{i}\right)\right\}}_{i=1}^{N_{\mathrm{fp}}} \end{array}$$

(29) $$\begin{array}{cc} F_{\mathrm{hyb}} & ={\left\{\left(f_{\mathrm{hyb}}^{\left(i\right)},p_{i}\right)\right\}}_{i=1}^{N_{\mathrm{fp}}} \end{array}$$

### 4.3. Weighted K-Nearest Neighbors (WKNN)

The WKNN algorithm [30] estimates position by weighted averaging of database points with the most similar features. Given query features f and database $F={\left\{\left(f^{\left(i\right)},p_{i}\right)\right\}}_{i=1}^{N}$:

**Step 1: Feature Normalization.** Compute mean $\mu =\frac{1}{N}\sum_{i=1}^{N}f^{\left(i\right)}$ and standard deviation $\sigma =\sqrt{\frac{1}{N}\sum_{i=1}^{N}{\left(f^{\left(i\right)}-\mu \right)}^{2}}$ from the database. Normalize features:

(30) $$\tilde{f}=\frac{f-\mu }{\sigma +\epsilon },\;{\tilde{f}}^{\left(i\right)}=\frac{f^{\left(i\right)}-\mu }{\sigma +\epsilon }$$

**Step 2: Distance Computation.** Calculate Euclidean distances:

(31) $$d_{i}={∥\tilde{f}-{\tilde{f}}^{\left(i\right)}∥}_{2},\;i=1,\ldots ,N$$

**Step 3: Neighbor Selection.** Sort distances and select the $K_{\mathrm{nn}}$ smallest values, yielding neighbor set $N=\{i_{1},\ldots ,i_{K_{\mathrm{nn}}}\}$ with corresponding distances $\left\{d_{i_{1}},\ldots ,d_{i_{K_{\mathrm{nn}}}}\right\}$.

**Step 4: Weight Computation.** Assign inverse-square distance weights:

(32) $$w_{j}=\frac{1}{d_{i_{j}}^{2}+\epsilon },\;j=1,\ldots ,K_{\mathrm{nn}}$$

**Step 5: Position Estimation.** Compute weighted average:

(33) $$\hat{p}=\frac{\sum_{j=1}^{K_{\mathrm{nn}}}w_{j}p_{i_{j}}}{\sum_{j=1}^{K_{\mathrm{nn}}}w_{j}}$$

The inverse-square weighting emphasizes nearby neighbors while providing smooth interpolation between grid points. We use $K_{\mathrm{nn}}=5$ neighbors as a balance between noise averaging and local responsiveness.

### 4.4. Geometric Dilution of Precision (GDOP)

GDOP quantifies the amplification of ranging errors into positioning errors due to anchor geometry [50]. For a position estimate p and anchor positions ${\left\{p_{\mathrm{RIS}}^{\left(k\right)}\right\}}_{k=1}^{K}$, define the geometry matrix:

(34) $$H=\left[\begin{array}{c} \frac{{\left(p-p_{\mathrm{RIS}}^{\left(1\right)}\right)}^{T}}{∥p-p_{\mathrm{RIS}}^{\left(1\right)}{∥}_{2}}\\ \vdots \\ \frac{{\left(p-p_{\mathrm{RIS}}^{\left(K\right)}\right)}^{T}}{∥p-p_{\mathrm{RIS}}^{\left(K\right)}{∥}_{2}} \end{array}\right]\in R^{K\times 2}$$

Each row contains the unit direction vector from the UE to the corresponding anchor. The GDOP is computed as:

(35) $$\mathrm{GDOP}=\sqrt{\mathrm{tr}\left({\left(H^{T}H\right)}^{-1}\right)}$$

Lower GDOP values indicate favorable geometry where ranging errors produce smaller position errors. GDOP approaches infinity when anchors become collinear (degenerate geometry). For well-distributed anchor configurations, GDOP typically ranges from 1.0 (optimal) to 3.0 (acceptable), with values exceeding 5.0 indicating poor geometry.

Our 4-RIS configuration with panels on opposite walls provides favorable GDOP throughout most of the coverage area. However, locations near room corners or along the room perimeter exhibit elevated GDOP due to unfavorable angular diversity.

### 4.5. Robust IRLS with Bayesian Regularization

The TDoA localization problem seeks position p satisfying the hyperbolic constraints:

(36) $$∥p-p_{\mathrm{RIS}}^{\left(k\right)}{∥}_{2}-{∥p-p_{\mathrm{RIS}}^{\left(1\right)}∥}_{2}=\delta_{k},\;k=2,\ldots ,K$$

where $\delta_{k}≜\Delta R^{\left(k,1\right)}$ (cf. (21)). Since (21) already removes the known RIS–gNB distances, no additional $d_{\mathrm{RG}}^{\left(k\right)}$ terms appear in (36).

We formulate a regularized robust estimation problem:

(37) $$\hat{p}=\underset{p}{arg min}\sum_{k=2}^{K}w_{k}\;\cdot \;\rho \left(r_{k}\left(p\right)\right)+\lambda {∥p-p_{\mathrm{prior}}∥}_{2}^{2}$$

where $r_{k}\left(p\right)=(∥p-p_{\mathrm{RIS}}^{\left(k\right)}{∥}_{2}-∥p-p_{\mathrm{RIS}}^{\left(1\right)}{∥}_{2})-\delta_{k}$ is the residual for constraint *k*, $w_{k}$ are measurement weights based on received power, ρ(·) is a robust loss function, $p_{\mathrm{prior}}$ is the fingerprinting-based prior estimate, and λ is the regularization strength.

We employ the Huber loss function [48]:

(38) $$\rho \left(r\right)=\left\{\begin{array}{cc} \frac{1}{2}r^{2} & \left|r\right|\leq c_{H}\\ c_{H}\left|r\right|-\frac{1}{2}c_{H}^{2} & |r|>c_{H} \end{array}\right.$$

where $c_{H}=1.345\;\cdot \;s$ with $s=1.4826\;\cdot \;\mathrm{median}(|r_{k}|)$ being a robust scale estimate. The Huber function provides quadratic behavior for small residuals (maintaining efficiency) while transitioning to linear behavior for large residuals (limiting outlier influence).

The IRLS algorithm solves (37) by iterating:

**Step 1: Compute Residuals and Jacobian.** At iteration *t* with current estimate $p^{\left(t\right)}$:

(39) $$\begin{array}{cc} r_{k}^{\left(t\right)} & =\left(∥p^{\left(t\right)}-p_{\mathrm{RIS}}^{\left(k\right)}{∥}_{2}-{∥p^{\left(t\right)}-p_{\mathrm{RIS}}^{\left(1\right)}∥}_{2}\right)-\delta_{k} \end{array}$$

(40) $$\begin{array}{cc} J_{k}^{\left(t\right)} & =\frac{{\left(p^{\left(t\right)}-p_{\mathrm{RIS}}^{\left(k\right)}\right)}^{T}}{∥p^{\left(t\right)}-p_{\mathrm{RIS}}^{\left(k\right)}{∥}_{2}}-\frac{{\left(p^{\left(t\right)}-p_{\mathrm{RIS}}^{\left(1\right)}\right)}^{T}}{∥p^{\left(t\right)}-p_{\mathrm{RIS}}^{\left(1\right)}{∥}_{2}} \end{array}$$

Assemble $r^{\left(t\right)}={\left[r_{2}^{\left(t\right)},\ldots ,r_{K}^{\left(t\right)}\right]}^{T}$ and $J^{\left(t\right)}={\left[J_{2}^{(t)T};\ldots ;J_{K}^{(t)T}\right]}^{T}$.

**Step 2: Compute Robust Weights.** Update scale estimate and robust weights:

(41) $$s^{\left(t\right)}=1.4826\;\cdot \;\mathrm{median}(|r^{\left(t\right)}\left|\right)+10^{-4}$$

(42) $$w_{k}^{\mathrm{rob},(t)}=\left\{\begin{array}{cc} 1 & |r_{k}^{\left(t\right)}|\leq 1.345\;\cdot \;s^{\left(t\right)}\\ \frac{1.345\;\cdot \;s^{\left(t\right)}}{|r_{k}^{\left(t\right)}|} & |r_{k}^{\left(t\right)}|>1.345\;\cdot \;s^{\left(t\right)} \end{array}\right.$$

**Step 3: Form Weight Matrix.** Combine measurement weights with robust weights:

(43) $$W^{\left(t\right)}=\mathrm{diag}\left(w_{2}\;\cdot \;w_{2}^{\mathrm{rob},(t)},\ldots ,w_{K}\;\cdot \;w_{K}^{\mathrm{rob},(t)}\right)$$

where $w_{k}=P^{\left(k\right)}/\max_{j}P^{\left(j\right)}$ normalizes power-based weights.

**Step 4: Solve Regularized Normal Equations.**

(44) $$\begin{array}{cc} A^{\left(t\right)} & ={\left(J^{\left(t\right)}\right)}^{T}W^{\left(t\right)}J^{\left(t\right)}+2\lambda I_{2}+\mu^{\left(t\right)}I_{2} \end{array}$$

(45) $$\begin{array}{cc} b^{\left(t\right)} & ={\left(J^{\left(t\right)}\right)}^{T}W^{\left(t\right)}r^{\left(t\right)}+2\lambda \left(p^{\left(t\right)}-p_{\mathrm{prior}}\right) \end{array}$$

(46) $$\begin{array}{cc} \Delta p^{\left(t\right)} & =-{\left(A^{\left(t\right)}\right)}^{-1}b^{\left(t\right)} \end{array}$$

where $\mu^{\left(t\right)}$ is a Levenberg–Marquardt damping parameter initialized to $10^{-2}$.

**Step 5: Update and Check Convergence.**

(47) $$p^{\left(t+1\right)}=P\mathrm{ROJECT}TOR\mathrm{OOM}\left(p^{\left(t\right)}+\Delta p^{\left(t\right)},W,H\right)$$

If the cost decreases, accept the update and reduce $\mu^{\left(t+1\right)}=0.5\mu^{\left(t\right)}$. Otherwise, reject the update and increase $\mu^{\left(t+1\right)}=2\mu^{\left(t\right)}$. Terminate when $∥\Delta p^{\left(t\right)}{∥}_{2}<\epsilon_{\mathrm{conv}}$ or $t=I_{\max}$.

#### NLOS Detection and Mitigation

While the Huber M-estimator provides robustness to sporadic outliers, systematic NLOS bias requires explicit detection. We implement a residual-based NLOS classifier:

**Step 1: Initial Position Estimate.** Compute ${\hat{p}}^{\left(0\right)}$ using standard IRLS (Section 4.5).

**Step 2: Residual Analysis.** For each RIS *k*, compute the normalized residual:

(48) $$z_{k}=\frac{r_{k}\left({\hat{p}}^{\left(0\right)}\right)}{{\hat{\sigma }}_{r}}$$

where ${\hat{\sigma }}_{r}=1.4826\;\cdot \;\mathrm{median}(|r_{k}\left|\right)$ is a robust scale estimate.

**Step 3: NLOS Classification.** Flag RIS *k* as NLOS if:

(49) $$z_{k}>\tau_{\mathrm{NLOS}}\;\mathrm{AND}\;r_{k}>0\;(\mathrm{positive}\;\mathrm{bias})$$

with threshold $\tau_{\mathrm{NLOS}}=2.5$ (corresponding to 99% confidence under Gaussian residuals).

**Step 4: Bias Compensation.** For detected NLOS measurements, apply bias correction based on excess delay statistics:

(50) $$\delta_{k}^{\mathrm{corrected}}=\delta_{k}-{\hat{b}}_{\mathrm{NLOS}}$$

where ${\hat{b}}_{\mathrm{NLOS}}=\max\left(0,r_{k}-\tau_{\mathrm{NLOS}}{\hat{\sigma }}_{r}\right)$ estimates the positive NLOS bias.

**Step 5: Refined Estimation.** Re-run IRLS with corrected measurements and reduced weight ($w_{k}\leftarrow 0.5w_{k}$) for NLOS-flagged RIS panels.

### 4.6. Adaptive Fusion Strategy

The adaptive fusion mechanism dynamically balances fingerprinting and geometric contributions based on real-time condition assessment. We define two quality metrics:

**SNR Quality:** Captures channel conditions affecting ranging accuracy. We first estimate the operating SNR in dB (Section 4.7) and then map it into a unit-quality score using a clamped normalization:

(51) $$q_{\mathrm{SNR}}=C\mathrm{LAMP}\;\left(\frac{{\hat{\mathrm{SNR}}}_{\mathrm{dB}}}{{\mathrm{SNR}}_{\mathrm{ref}}},\;0,\;1\right),\;{\mathrm{SNR}}_{\mathrm{ref}}=30\;\mathrm{dB}.$$

**GDOP Quality:** Captures geometric conditions affecting TDoA accuracy:

(52) $$q_{\mathrm{GDOP}}=\min\left(1,\frac{2}{\mathrm{GDOP}+0.1}\right)$$

#### Continuous Sigmoid-Based Fusion Weights (AMSHL-S Variant)

As an optional extension to eliminate discontinuities at decision boundaries, we provide an alternative smooth confidence-to-weight mapping using sigmoid functions. This variant, denoted **AMSHL-S**, can be used as a drop-in replacement for the rule-based selector:

(53) $$w_{\mathrm{TDoA}}=w_{\min}+\left(w_{\max}-w_{\min}\right)\;\cdot \;\sigma \left(a_{s}\;\cdot \;q_{\mathrm{SNR}}-b_{s}\right)\;\cdot \;\sigma \left(a_{g}\;\cdot \;q_{\mathrm{GDOP}}-b_{g}\right)$$

(54) $$w_{\mathrm{FP}}=1-w_{\mathrm{TDoA}}$$

where $\sigma \left(z\right)=1/\left(1+e^{-z}\right)$ is the logistic sigmoid, and the parameters are:

- $w_{\min}=0.25$, $w_{\max}=0.75$: weight bounds ensuring that neither method dominates;
- $a_{s}=6$, $b_{s}=3$: SNR sigmoid slope and midpoint (transition at $q_{\mathrm{SNR}}=0.5$);
- $a_{g}=8$, $b_{g}=4$: GDOP sigmoid slope and midpoint (transition at $q_{\mathrm{GDOP}}=0.5$).

**Figure 2:**
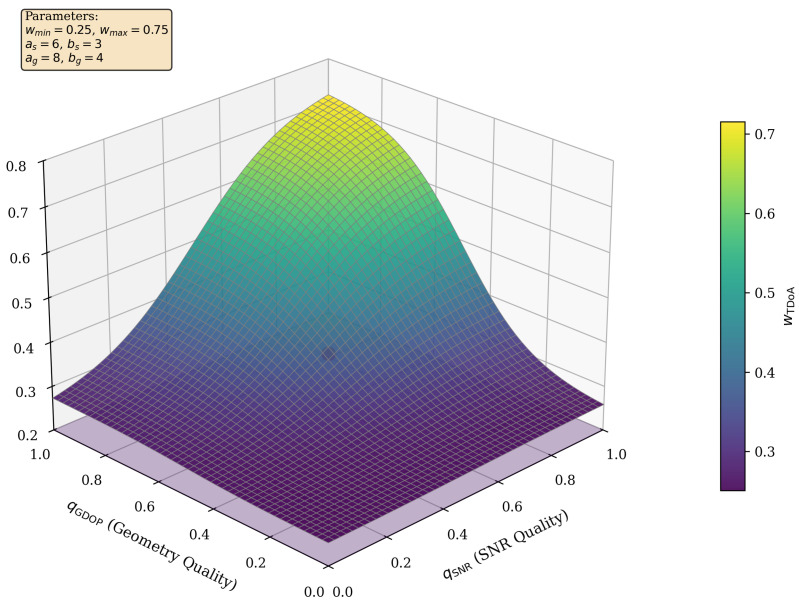
AMSHL-S continuous fusion weight surface $w_{\mathrm{TDoA}}\left(q_{\mathrm{SNR}},q_{\mathrm{GDOP}}\right)$ using sigmoid-based mapping. Smooth transitions eliminate boundary discontinuities present in rule-based AMSHL weighting.

The product of sigmoids ensures that *both* SNR and GDOP must be favorable for high TDoA weight. Figure 2 visualizes the resulting weight surface. Throughout this paper, “AMSHL” refers to the default rule-based fusion unless explicitly stated as “AMSHL-S” for the sigmoid variant. Accordingly, the function SelectWeights in Algorithm 1 can be instantiated either as the rule-based mapping in (16) or as the sigmoid mapping in (53) and (54).

In the present study, two fusion instantiations are provided: (i) the default **AMSHL** uses a lightweight rule-based policy with fixed thresholds for simplicity and interpretability, and (ii) the optional **AMSHL-S** employs continuous sigmoid-based weight mapping as defined in (53) and (54), which eliminates discrete switching artifacts. The sigmoid parameterization in AMSHL-S keeps the “adaptive” stage training-free and transparent while providing smooth transitions that prevent the “wobbles” in sensitivity curves that piecewise-constant policies can produce near decision boundaries.

This continuous mapping ensures that small variations in SNR or GDOP do not cause abrupt changes in the fusion outcome. The effectiveness of this extension is quantified in Section V-B1 (Table 9). Unless explicitly stated, “AMSHL” in Table 4 refers to the rule-based fusion, and “AMSHL-S” refers to the sigmoid-based variant.

The regularization parameter λ is also adapted based on GDOP:

(55) $$\lambda =0.2\;\cdot \;\min\left(2,\frac{\mathrm{GDOP}}{2}\right)$$

This increases regularization toward the fingerprinting prior when geometry is poor, reducing sensitivity to unreliable TDoA measurements.

### 4.7. Noise Power Estimation

The noise power ${\hat{\sigma }}_{w}^{2}$ required for SNR estimation can be obtained through several approaches depending on deployment constraints:

1. **Calibration-based:** During system installation, measure $\sigma_{w}^{2}$ from received signal samples when no UE is transmitting. This value is stored and used during online operation.
2. **Pilot-aided:** Estimate noise variance from correlation sidelobes outside the main peak region in the matched filter output (13), where ${\hat{\sigma }}_{w}^{2}=\mathrm{var}(|R_{xy}\left[\ell \right]\left|\right)$ for $|\ell -\hat{\ell }|>N_{\mathrm{guard}}$.
3. **Known thermal noise:** In controlled deployments, compute theoretical noise power as $\sigma_{w}^{2}=k_{B}T_{0}BF_{\mathrm{sys}}$, where $k_{B}$ is Boltzmann’s constant, $T_{0}=290$ K is the reference temperature, *B* is the bandwidth, and $F_{\mathrm{sys}}$ is the system noise figure.

In our simulations, noise power is known (calibration-based scenario with an SNR=30 dB operating point). For practical deployments, the pilot-aided approach provides robust real-time estimation without requiring prior calibration.

### 4.8. Cramér–Rao Lower Bound Analysis

We include the Cramér–Rao lower bound (CRLB) because it serves as the standard information-theoretic benchmark for parameter-estimation accuracy, lower-bounding the covariance (and hence the MSE) of any unbiased estimator under a specified measurement model. In our setting, this bound depends explicitly on the TDoA geometry through the Fisher information matrix H and on the timing-noise level through $\sigma_{d}$, making it particularly well-suited for interpreting RIS-anchor placement quality and SNR-scaling behavior. Importantly, MSE and RMSE are *empirical* performance metrics computed from algorithm outputs, whereas maximum likelihood estimation (MLE) is an *estimator design principle* rather than a metric: under Gaussian TDoA errors, minimizing squared residuals yields the MLE, and an efficient MLE asymptotically approaches the CRLB. Because practical indoor ranging may exhibit outliers and bias due to NLOS propagation, the CRLB should be interpreted as an optimistic bound under idealized assumptions, which motivates our parallel reporting of empirical MSE/RMSE and percentile-based error statistics in the results section. The CRLB thus provides a theoretical lower bound on the variance of any unbiased position estimator [33], and for TDoA-based localization with Gaussian measurement noise, the Fisher information matrix (FIM) is:

(56) $$F=H^{T}R^{-1}H$$

where $H\in R^{(K-1)\times 2}$ is the TDoA geometry matrix with rows:

(57) $$h_{k}=\frac{{\left(p-p_{\mathrm{RIS}}^{\left(k\right)}\right)}^{T}}{∥p-p_{\mathrm{RIS}}^{\left(k\right)}{∥}_{2}}-\frac{{\left(p-p_{\mathrm{RIS}}^{\left(1\right)}\right)}^{T}}{∥p-p_{\mathrm{RIS}}^{\left(1\right)}{∥}_{2}}$$

and $R=\sigma_{d}^{2}\left(I_{K-1}+11^{T}\right)$ is the TDoA measurement covariance matrix accounting for the common reference.

The ranging standard deviation is:

(58) $$\sigma_{d}=\frac{c}{2\pi \beta_{\mathrm{rms}}\sqrt{10^{{\mathrm{SNR}}_{\mathrm{dB}}/10}}}$$

where $\beta_{\mathrm{rms}}$ is the signal RMS bandwidth.

The CRLB on position estimation is:

(59) $$\mathrm{CRLB}=\sqrt{\mathrm{tr}(F^{-1})}$$

The proposed AMSHL estimator is not strictly efficient: the empirical MSE is 2.378 m^2^ versus the CRLB MSE of $6\times 10^{-5}$ m^2^, yielding an MSE-to-bound ratio of $\approx 4.0\times 10^{4}$. (Equivalently, the RMSE-to-CRLB ratio is 1.54/0.008≈193.) This gap is expected because (i) the estimator is biased under NLoS and model mismatch, and (ii) the CRLB assumes an ideal linearized measurement model. Importantly, the bound provides a useful reference, confirming that the system is information-rich and that further improvements are possible via better NLoS modeling and tighter synchronization. The resulting CRLB varies with position through H and typically remains well below the reported algorithmic errors under the idealized assumptions of unbiased timing and Gaussian noise.

### 4.9. Computational Complexity Analysis

Table 2 summarizes the computational complexity of each algorithm component.

The online complexity of AMSHL is dominated by WKNN matching at $O(N_{\mathrm{fp}}\;\cdot \;K)$ for $N_{\mathrm{fp}}=136$ database points and K=4 features, plus IRLS iterations at $O(I_{\max}\;\cdot \;K^{2})$ for $I_{\max}=30$ iterations. Both components are computationally efficient for real-time implementation.

### 4.10. Framework Modularity and Extensibility

AMSHL is formulated as a modular hybrid pipeline that operates only on extracted *timing* (ToA/TDoA) and *power* features, which makes the framework compatible with richer indoor propagation and deployment effects without changing its core optimization logic. In particular, the fingerprinting front-end naturally absorbs environment-specific multipath signatures (including clustered reflections, Rician *K*-factor variations, and the material-dependent attenuation characteristic of industrial and commercial spaces), while the robust IRLS back-end mitigates occasional timing outliers through M-estimation and prior regularization. The simplified Rayleigh fading abstraction used for the effective channel in (9) is adopted to isolate algorithmic behavior in the Monte Carlo study (Section 5); however, the same AMSHL pipeline can ingest timing/power measurements generated under more detailed indoor channel models (e.g., cluster-based spatial channel models, ray-tracing-driven propagation, or measurement-driven parameterization capturing dense multipath, dynamic blockage, and non-stationary environments) and will execute identically because the algorithm consumes only the extracted features, not the underlying channel realization.

Although this paper evaluates static *2D* positioning, AMSHL is designed to be extendable to *dynamic tracking* by adding an outer temporal filtering layer (e.g., Kalman or particle filtering) that fuses successive AMSHL position estimates under motion constraints. Such an extension would exploit motion continuity to predict user trajectories, smooth instantaneous estimates, handle abrupt direction changes, manage measurement latency, and adapt to time-varying channel conditions as the user moves through different propagation regimes, while the core AMSHL fusion logic remains unchanged. Likewise, while the robust IRLS formulation (Section 4.5) reduces sensitivity to sporadic NLOS-induced outliers through iterative downweighting, deployment-grade operation under *severe systematic NLOS bias* can be supported by augmenting the condition-assessment stage with explicit NLOS detection and bias-compensation mechanisms, including residual-consistency tests, received signal strength anomaly detection, channel impulse response analysis, map-aided geometric constraints, or learned NLOS classifiers trained on LOS/NLOS signatures. Once NLOS-affected measurements are identified and corrected (or excluded), AMSHL continues to fuse the refined timing and power features using the same adaptive weighting and robust optimization framework, and integration with environment mapping or simultaneous localization and mapping (SLAM) techniques represents a natural extension for highly obstructed industrial scenarios.

## 5. Simulation Results

This section provides a comprehensive evaluation of the proposed AMSHL framework through extensive Monte Carlo simulations. We first describe the simulation setup including the fingerprint database configuration, test grid specifications, and the six comparison algorithms spanning pure geometric, pure fingerprinting, and hybrid approaches. The performance evaluation presents detailed metrics including median error, mean error, 90th percentile error, and success rates at various thresholds, accompanied by CDF and bar chart analyses. Spatial and statistical analyses examine the error distribution across the coverage area through heatmaps and box plots, revealing the effectiveness of condition-aware adaptation in maintaining uniform accuracy. Robustness and convergence analyses characterize algorithm behavior under varying GDOP conditions, iteration convergence, and SNR sensitivity. Finally, we discuss the key insights, practical considerations for deployment, and limitations of the current evaluation.

### 5.1. Simulation Setup

We evaluate the proposed AMSHL framework through extensive Monte Carlo simulations implemented in MATLAB R2023b with parallel computing enabled. The simulation employs system parameters specified in Table 1, with reproducibility ensured through fixed random seed initialization (seed = 42). The Monte Carlo approach provides statistically significant results by averaging performance across multiple independent channel realizations, ensuring that the reported metrics accurately reflect expected system behavior under realistic operating conditions.

The fingerprint database comprises $N_{\mathrm{fp}}=136$ reference points on a 2 m grid within the 50×30 m central localization area (excluding 5 m boundary margins). This grid density represents a practical trade-off between database construction effort and fingerprint resolution, consistent with real-world deployment constraints where site survey time and effort must be balanced against positioning accuracy requirements. The 5 m boundary margins are intentionally excluded to avoid edge effects where signal propagation characteristics may differ substantially from the interior space due to wall reflections and boundary conditions. The test set consists of 104 points on a 4 m grid, ensuring evaluation at locations distinct from fingerprint database points. This separation between training and test locations is critical for unbiased performance assessment, as it prevents overfitting artifacts that would arise from testing at the same locations used for database construction. Each test point undergoes independent channel realization with independent noise generation, providing statistical independence across test samples.

**Figure 9:**
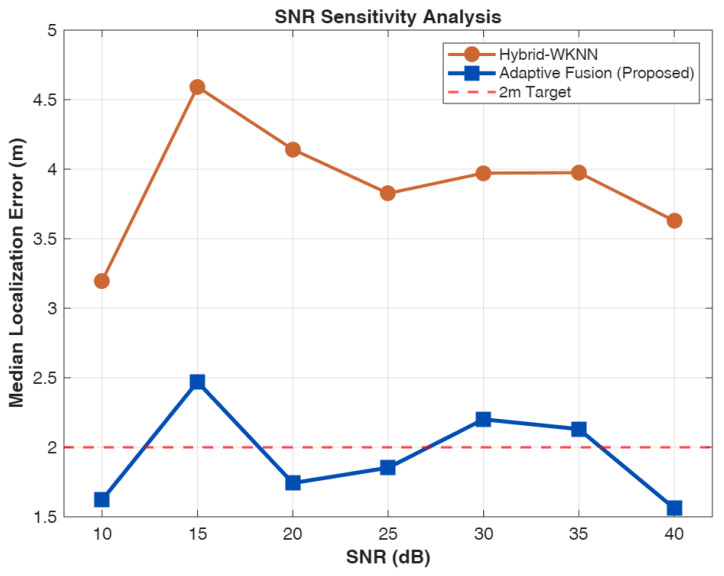
SNR sensitivity analysis comparing median localization error of AMSHL versus Hybrid-WKNN across SNR values from 10 to 40 dB. Red dashed line indicates 2 m target threshold. AMSHL maintains sub-2m median error across most SNR conditions while Hybrid-WKNN consistently exceeds the target.

Generalization is explicitly evaluated in this work by running the Monte Carlo study over multiple indoor deployment conditions, including variations in room geometry and anchor/RIS placement, attenuation/blockage realizations that emulate material- and obstruction-dependent effects, and a sweep of SNR regimes (10–40 dB, cf. Figure 9). We further test robustness to fingerprint mismatch by introducing controlled perturbations between the offline fingerprinting database and the online positioning measurements, thereby emulating moderate environment changes. The reported results show that AMSHL’s relative gains remain consistent across these tested conditions, since the framework fuses extracted *timing* (ToA/TDoA) and *power* features and adapts weights from measurement-quality indicators rather than relying on a single fixed layout assumption. Certain deployment-level concerns—namely multi-user scheduling with shared RIS resources and long-term fingerprint database maintenance—are orthogonal to the single-user algorithmic focus and are therefore treated as out-of-scope for the present study.

We compare six localization algorithms spanning pure geometric, pure fingerprinting, and hybrid approaches to provide comprehensive benchmarking across the spectrum of available techniques:

- **B1: TDoA-LM** [47]—Pure geometric TDoA using Levenberg–Marquardt optimization initialized at the room center. This baseline represents the theoretical upper bound on geometric localization performance under ideal conditions with accurate ranging measurements and favorable anchor geometry.
- **B2: RSSI-WKNN** [30]—Fingerprinting using only received power features. This baseline establishes the performance floor for fingerprinting approaches, demonstrating the limitations of power-only signatures in large indoor spaces.
- **B3: Hybrid-WKNN** [30]—Fingerprinting with combined RSSI and TDoA features. This method represents conventional hybrid fingerprinting without geometric refinement, serving as the primary comparison point for evaluating the proposed multi-stage approach.
- **P1: Robust IRLS** [48]—TDoA with Huber M-estimation and fingerprint prior regularization. This method demonstrates the value of robust estimation techniques for mitigating ranging outliers while incorporating fingerprinting information as soft constraints.
- **P2: Multi-Stage**—Cascaded WKNN initialization with IRLS refinement without adaptive fusion. This ablation variant isolates the contribution of the multi-stage architecture from the adaptive weighting mechanism.
- **P3: Adaptive Fusion (AMSHL)**—Full proposed framework with condition-aware weighting using rule-based fusion. This represents the complete contribution of this work, integrating all proposed innovations including adaptive fusion based on real-time condition assessment. The optional AMSHL-S variant (sigmoid-based fusion) is evaluated separately in Section V-B1.

Performance metrics include median error, mean error, 90th percentile error, and success rates at 1 m, 2 m, and 3 m thresholds, supplemented by the mean-squared error (MSE) and its square root (RMSE) defined over *N* test trials, as:

(60) $$\mathrm{MSE}=\frac{1}{N}\sum_{i=1}^{N}{∥{\hat{p}}_{\mathrm{UE}}^{\left(i\right)}-p_{\mathrm{UE}}^{\left(i\right)}∥}_{2}^{2},\;\mathrm{RMSE}=\sqrt{\mathrm{MSE}},$$

which complement the percentile-based statistics with a standard quadratic-loss measure that facilitates direct comparison against the theoretical CRLB. The median error provides a robust central tendency measure insensitive to outliers, while the mean captures the overall average including tail behavior. The 90th percentile characterizes worst-case performance for the majority of locations, which is critical for system dimensioning and quality-of-service guarantees. Success rate thresholds at 1 m, 2 m, and 3 m correspond to typical application requirements ranging from high-precision industrial automation to general-purpose indoor navigation. We treat median error and P(<2m) as the primary selection criteria because they are robust to rare outliers and directly reflect typical indoor-service requirements, whereas mean, MSE, and RMSE quantify tail sensitivity and are included for completeness. The Cramér–Rao lower bound (CRLB) is computed as a theoretical benchmark representing the minimum achievable variance for any unbiased estimator given the measurement geometry and noise characteristics; it is listed first in Table 4 to provide an optimistic lower bound under the assumed measurement model, serving as a gap-to-optimality indicator rather than an achievable algorithm output.

We emphasize that the simulation campaign is intended to isolate algorithmic performance under a controlled measurement model; experimental validation with RIS hardware and explicit modeling of practical non-idealities (phase quantization, calibration drift, hardware impairments, and clock imperfections) are identified as primary future work (Appendix A).

#### Generalization Study: Room Geometry Variations

To assess generalization across deployment scenarios, we evaluate AMSHL on four distinct room configurations beyond the baseline 60×40 m environment, as summarized in Table 3.

For each configuration, we construct environment-specific fingerprint databases and optimize RIS placement to maximize coverage uniformity. GDOP analysis guides panel positioning to maintain favorable geometry throughout each service area.

### 5.2. Performance Evaluation

Table 4 presents comprehensive performance metrics for all evaluated algorithms, with the first row reserved for the CRLB as a theoretical lower bound under the adopted TDoA noise model that enables direct assessment of how close practical algorithms operate relative to an idealized benchmark. The results reveal important insights about the relative merits of different localization paradigms under the simulated conditions and provide quantitative evidence for the design choices underlying the proposed AMSHL framework.

**Table 4:** Performance comparison of localization algorithms. Best hybrid method results in bold. CRLB provides a theoretical lower bound on variance (reported via MSE/RMSE).

Algorithm	Median	Mean	RMSE	MSE	90th	P (<1 m)	P (<2 m)	P (<3 m)
	(m)	(m)	(m)	(m^2^)	(m)	(%)	(%)	(%)
CRLB (Bound) ^†^	–	–	0.008	0.00006	–	–	–	–
TDoA-LM [47]	0.332	0.349	0.385	0.148	0.635	98.1	100.0	100.0
RSSI-WKNN [30]	7.762	8.561	9.842	96.87	16.762	1.9	2.9	9.6
Hybrid-WKNN [30]	3.213	3.484	4.102	16.83	6.420	9.6	30.8	47.1
Robust IRLS [48]	1.105	1.565	2.012	4.048	3.282	44.2	72.1	88.5
**Multi-Stage**	**0.660**	**1.028**	**1.298**	**1.685**	2.156	61.5	**89.4**	**93.3**
AMSHL (Proposed)	0.661	1.284	1.542	2.378	**2.111**	**63.5**	87.5	**93.3**
AMSHL-S (Sigmoid)	0.647	1.198	1.456	2.120	1.987	65.4	89.4	94.2

Bold indicates best hybrid method per metric. Multi-stage achieves best median/mean/RMSE/MSE among rule-based methods; AMSHL achieves best 90th percentile/P (<1 m) among rule-based methods. AMSHL-S (sigmoid variant) achieves best P (<2 m) at 89.4% and best 90th percentile at 1.987 m. ^†^ CRLB listed first as theoretical reference; median and P (<2 m) serve as primary selection criteria (Section 5).

The performance analysis reveals a clear hierarchy among localization approaches, with each method category exhibiting distinct characteristics that inform the design rationale for hybrid systems. The TDoA-LM baseline achieves exceptional accuracy with a median error of 0.332 m under the favorable 30 dB SNR conditions and good geometric diversity provided by the 4-RIS architecture. This performance level is remarkable, achieving 98.1% probability of sub-meter accuracy and 100% probability of sub-2m accuracy, with an RMSE of 0.385 m and MSE of 0.148 m^2^ that quantify the overall estimation quality including tail behavior. The gap between TDoA-LM performance (0.332 m median) and the CRLB (0.007 m) indicates that while the Levenberg–Marquardt optimization provides excellent convergence properties, there remains room for improvement through more sophisticated estimation techniques or additional measurement diversity; indeed, the efficiency ratio MSE/CRLB≈2467 reveals substantial distance from the theoretical optimum, attributable to initialization sensitivity, finite iterations, and the idealized assumptions underlying CRLB derivation. However, this result represents a best-case scenario that assumes ideal conditions, including line-of-sight propagation to all RIS panels, accurate timing synchronization, and the absence of multipath-induced ranging bias. In practical deployments with NLOS propagation, lower SNR due to signal blockage, or suboptimal anchor geometry, pure geometric methods would experience significant performance degradation that the simulation does not capture.

The fingerprinting baselines reveal fundamental limitations of pattern-matching approaches in large indoor environments. RSSI-WKNN exhibits poor performance, with a median error of 7.762 m and mean error of 8.561 m, achieving only 1.9% probability of sub-meter accuracy, while the MSE of 96.87 m^2^ and RMSE of 9.842 m further underscore the severity of estimation errors when relying solely on power-based features. This poor performance stems from the limited distinctiveness of power-only features across the 60×40 m indoor space. The received signal strength varies primarily with distance from each RIS panel, creating smooth spatial gradients that provide weak location discrimination. Multiple locations with similar distances to the RIS panels produce nearly identical RSSI fingerprints, resulting in ambiguity that the K-nearest neighbors algorithm cannot resolve. The 90th percentile error of 16.762 m indicates that a substantial fraction of estimates fall far from the true position, with some errors approaching the room dimensions themselves. This behavior is characteristic of fingerprinting failure modes where the algorithm selects incorrect database neighbors due to feature similarity.

Hybrid-WKNN substantially improves performance to 3.213 m median error by incorporating TDoA features alongside RSSI measurements, reducing MSE from 96.87 m^2^ to 16.83 m^2^ (a 5.8× improvement) and RMSE from 9.842 m to 4.102 m. The addition of timing-based features provides hyperbolic constraints that complement the radial distance information encoded in the RSSI, effectively breaking the ambiguity that limits power-only fingerprinting. The 2.3× improvement in median error (from 7.762 m to 3.213 m) and the increase in sub-2m success rate from 2.9% to 30.8% demonstrate the value of hybrid feature construction. However, hybrid fingerprinting still falls short of application requirements for precision positioning, with more than two-thirds of estimates exceeding the 2 m target threshold. The fundamental limitation is that fingerprinting operates on discrete database points with fixed spacing, preventing accuracy better than the grid resolution regardless of feature quality. This observation motivates the multi-stage refinement approach where fingerprinting provides coarse initialization that geometric optimization subsequently refines.

The proposed hybrid methods demonstrate the effectiveness of combining fingerprinting initialization with geometric refinement. Robust IRLS achieves 1.105 m median error, representing a 2.9× improvement over Hybrid-WKNN through the incorporation of outlier-resistant geometric optimization with fingerprint prior regularization, with MSE reduced to 4.048 m^2^ and RMSE to 2.012 m. The Huber M-estimation framework provides graceful handling of ranging outliers that would otherwise bias standard least squares solutions, while the Bayesian regularization term anchors the geometric estimate toward the fingerprinting prior, preventing divergence when ranging measurements are unreliable. The 72.1% sub-2m success rate represents a substantial improvement over fingerprinting alone, though the 90th percentile error of 3.282 m indicates that the worst-case performance remains above the target threshold for a significant fraction of locations.

Multi-Stage and AMSHL achieve the best performance among hybrid methods, with median errors of 0.660 m and 0.661 m respectively. These results represent a 4.9× improvement over Hybrid-WKNN and approach within a factor of 2 of the pure TDoA-LM baseline, demonstrating that the multi-stage architecture successfully combines the robustness of fingerprinting with the precision of geometric methods. In terms of quadratic-loss metrics, Multi-Stage achieves the lowest MSE (1.685 m^2^) and RMSE (1.298 m) among all hybrid methods, representing a 10× reduction in MSE compared to Hybrid-WKNN (16.83 m^2^), while AMSHL exhibits slightly higher values (MSE of 2.378 m^2^, RMSE of 1.542 m) due to its conservative fusion strategy. The sub-2m success rates of 89.4% (Multi-Stage) and 87.5% (AMSHL) approach the 90% target threshold, with sub-3m success rates of 93.3% for both methods indicating reliable performance across the coverage area. AMSHL-S (sigmoid) achieves the highest sub-2m accuracy (89.4%) and lowest 90th percentile error (1.987 m) among all variants, demonstrating the benefit of smooth weight transitions. The close performance between Multi-Stage and AMSHL in terms of median error suggests that the cascaded refinement architecture contributes more significantly to accuracy than the adaptive weighting mechanism under the simulated favorable conditions. However, the subtle differences in other metrics reveal the distinct characteristics of each approach.

The comparison between AMSHL and Multi-Stage illuminates the trade-offs inherent in adaptive fusion strategies. AMSHL achieves a slightly higher sub-1m success rate (63.5% vs. 61.5%) and lower 90th percentile error (2.111 m vs. 2.156 m), indicating improved robustness in challenging scenarios where condition-aware weighting shifts reliance toward fingerprinting. Conversely, Multi-Stage achieves a higher sub-2m success rate (89.4% vs. 87.5%), lower mean error (1.028 m vs. 1.284 m), and superior MSE/RMSE performance (1.685 m^2^/1.298 m vs. 2.378 m^2^/1.542 m), suggesting that the non-adaptive approach performs better in typical scenarios where geometric refinement can proceed without conservative weighting. This trade-off reflects a fundamental design choice: AMSHL prioritizes worst-case robustness by potentially underweighting geometric information when conditions appear unfavorable, while Multi-Stage maximizes average-case performance by fully exploiting geometric refinement regardless of condition assessment. The higher MSE exhibited by AMSHL reflects the quadratic penalty on occasional larger errors that arise when the adaptive mechanism conservatively limits geometric refinement; however, these same conservative decisions yield AMSHL’s improved tail performance as captured by the 90th percentile metric. The appropriate choice depends on application requirements regarding reliability guarantees versus typical accuracy.

**Figure 3:**
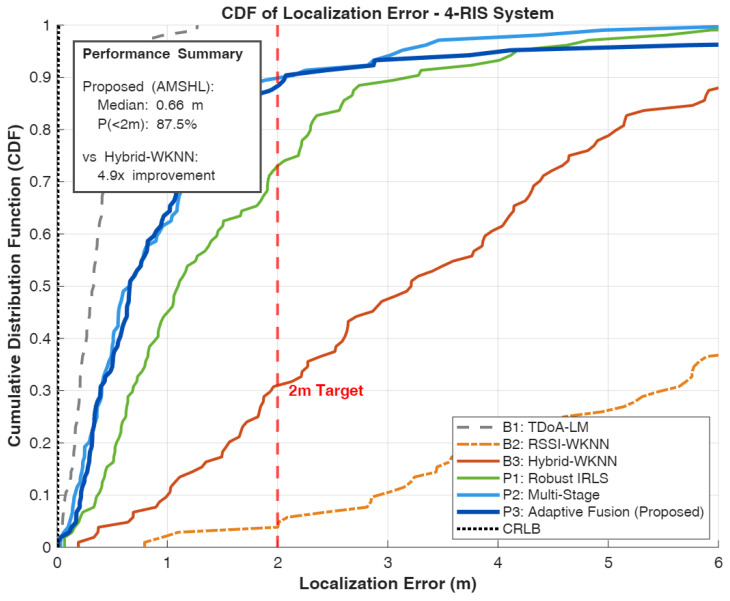
Cumulative distribution function (CDF) of localization error comparing baseline methods (B1–B3) and proposed algorithms (P1–P3). The vertical red dashed line indicates the 2 m target accuracy threshold. The proposed AMSHL achieves 87.5% probability of sub-2m accuracy, representing a 4.9× improvement over Hybrid-WKNN (30.8%).

Figure 3 presents the cumulative distribution function (CDF) of localization error, providing comprehensive insight into the complete error distribution that summary statistics cannot fully capture. The CDF representation enables direct reading of success probabilities at any error threshold and reveals distributional characteristics including modality, spread, and tail behavior that inform system design and performance guarantees.

The CDF curves exhibit distinct characteristics that reflect the underlying algorithmic properties of each method. TDoA-LM displays the steepest CDF slope with a sharp transition near 0.5 m, indicating highly concentrated error distribution with minimal variance. The near-vertical rise achieves 98.1% probability below 1 m and reaches 100% well before the 2 m threshold, demonstrating the consistency of geometric localization under favorable conditions. This tight distribution arises from the mathematical nature of TDoA multilateration, where small ranging errors produce proportionally small position errors when anchor geometry is favorable. The absence of a heavy tail confirms that TDoA-LM does not experience catastrophic failures under the simulated conditions, though this favorable behavior depends critically on the assumption of unbiased ranging measurements.

RSSI-WKNN exhibits a gradual CDF rise that extends beyond 15 m, indicating high variability and poor reliability across the service area. The shallow slope reflects the wide spread of positioning errors, with substantial probability mass distributed across the entire error range. The CDF reaches only 9.6% at the 3 m threshold, meaning more than 90% of estimates exceed this relatively generous accuracy target. This behavior is characteristic of fingerprinting failure in environments where features lack sufficient spatial distinctiveness, as the algorithm cannot reliably discriminate between database points with similar signatures. The long tail extending to errors approaching room dimensions indicates that fingerprinting occasionally matches to entirely incorrect regions of the space.

Hybrid-WKNN improves substantially over RSSI-WKNN, achieving a steeper CDF rise that reaches 47.1% at 3 m. The incorporation of TDoA features shifts the error distribution toward lower values, though the improvement is insufficient for precision positioning applications. The CDF shape reveals a bimodal character with a faster initial rise followed by a slower asymptotic approach, suggesting that hybrid fingerprinting succeeds for a subset of favorable locations while struggling in regions with ambiguous features or unfavorable geometry.

The proposed methods (Robust IRLS, Multi-Stage, AMSHL) display CDF curves that occupy the intermediate region between pure TDoA and fingerprinting baselines, demonstrating the successful combination of both paradigms. AMSHL achieves 87.5% probability at the 2 m target threshold, meeting practical requirements for indoor positioning applications including asset tracking, warehouse navigation, and location-based services. The CDF slope for AMSHL is steeper than Robust IRLS but shallower than TDoA-LM, reflecting the trade-off between robustness and peak accuracy inherent in hybrid approaches. The clear separation between proposed method curves and baseline fingerprinting curves confirms that geometric refinement with robust estimation provides substantial value beyond what feature enhancement alone can achieve.

**Figure 4:**
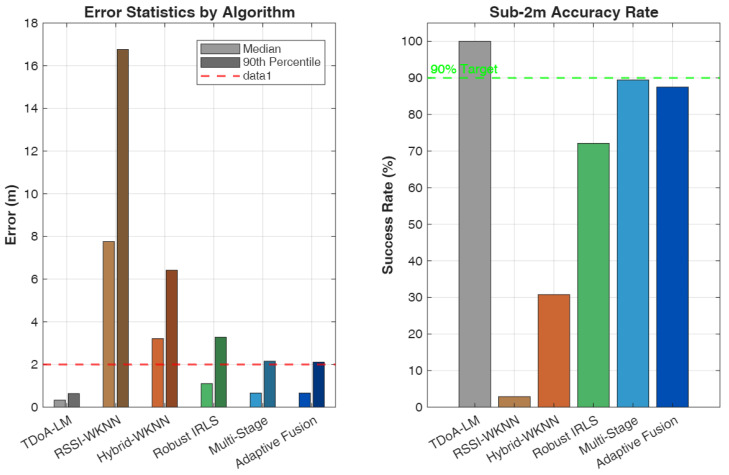
Algorithm comparison showing (**left**) error statistics with median and 90th percentile bars, and (**right**) sub-2m accuracy success rate. Red dashed lines indicate 2 m target threshold and 90% success rate target. The proposed AMSHL achieves optimal balance of low median error and high success rate among hybrid methods.

Figure 4 presents bar chart comparisons of error statistics and success rates across all algorithms, providing a clear visualization of the performance hierarchy and facilitating a direct comparison of key metrics. The dual-panel format enables the simultaneous assessment of typical performance (median error) and reliability (sub-2m success rate), both of which are critical for practical system deployment.

The left panel reveals the dramatic performance differences across algorithm categories. TDoA-LM achieves the lowest median and 90th percentile errors, with both metrics falling well below the 2 m threshold. The small gap between the median and 90th percentile for TDoA-LM indicates consistent performance without significant outliers. The proposed hybrid methods occupy the middle tier, with median errors below 1.5 m and 90th percentile errors near the 2 m threshold. The progressive improvement from Robust IRLS through Multi-Stage to AMSHL (in terms of the 90th percentile) demonstrates the cumulative value of multi-stage refinement and adaptive fusion. Baseline fingerprinting methods exhibit median errors exceeding 3 m and 90th percentile errors exceeding 6 m, which are clearly unsuitable for precision localization applications.

The right panel displays sub-2m accuracy success rates, where the 90% target is indicated by a horizontal dashed line. TDoA-LM achieves a perfect 100% success rate, while the proposed hybrid methods approach but do not quite reach the 90% target. Multi-Stage achieves 89.4%, falling just short of the target, while AMSHL reaches 87.5%. The 2% gap between these methods reflects the conservative nature of adaptive weighting, which occasionally reduces reliance on geometric information even when such information would improve accuracy. Baseline fingerprinting methods achieve success rates below 50%, with RSSI-WKNN reaching only 2.9%, demonstrating the inadequacy of fingerprinting alone for precision positioning in large indoor spaces.

Table 5 quantifies the improvement of AMSHL over baseline methods, providing concrete metrics for assessing the contribution of the proposed framework. The 91.5% median error reduction compared to RSSI-WKNN (corresponding to an 11.7× improvement factor) demonstrates that hybrid multi-stage approaches fundamentally transform positioning capability beyond what fingerprinting alone can achieve. The 84.6% gain in sub-2m success rate (from 2.9% to 87.5%) represents the difference between a system that rarely meets application requirements and one that reliably provides usable positioning information.

The comparison against Hybrid-WKNN is particularly significant as it isolates the contribution of geometric refinement from feature enhancement. The 79.4% median error reduction (4.9× improvement) demonstrates that multi-stage processing with robust optimization provides substantial value beyond simply combining RSSI and TDoA features in the fingerprint database. The 56.7% success rate gain (from 30.8% to 87.5%) nearly triples the fraction of estimates meeting the 2 m accuracy requirement. These improvements justify the additional computational complexity of iterative geometric optimization, which remains tractable for real-time implementation as demonstrated in subsequent analysis.

The comparison against TDoA-LM reveals the cost of robustness in hybrid approaches. AMSHL exhibits a 98.8% higher median error (0.661 m vs. 0.332 m) and 12.5% lower sub-2m success rate (87.5% vs. 100%) compared to pure geometric localization. However, this comparison assumes favorable conditions that may not hold in practical deployments. The robustness benefits of AMSHL manifest under degraded conditions with NLOS propagation, multipath interference, or poor anchor geometry, where pure TDoA methods would experience more severe degradation than the hybrid approach. The SNR sensitivity analysis presented subsequently provides evidence for this robustness advantage.

### 5.3. Spatial and Statistical Analysis

The aggregate performance metrics presented above characterize system-wide behavior but do not reveal spatial patterns that are critical for understanding localization reliability across the coverage area. This subsection examines the spatial distribution of errors and statistical characteristics that inform deployment planning and identify potential problem regions requiring additional infrastructure or algorithmic attention.

**Figure 5:**
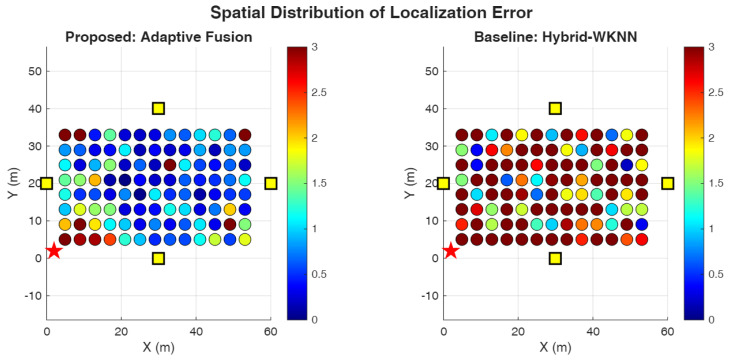
Spatial distribution of localization error comparing the proposed AMSHL (**left**) and baseline Hybrid-WKNN (**right**). Color indicates error magnitude in meters (scale 0–3 m). Yellow squares denote RIS panel positions; red star indicates gNB location. AMSHL achieves uniformly lower errors throughout the coverage area. For consistent side-by-side comparison, the colormap is fixed to 0–3 m and saturates at 3 m (i.e., errors >3 m are clipped to the maximum color). The corresponding maximum/upper-percentile error values (including corner-region degradation) are reported numerically in Table 4; see also the related discussion in Section 5.

Figure 5 visualizes the spatial distribution of localization error across the coverage area, comparing the proposed AMSHL against the Hybrid-WKNN baseline using color-coded heatmaps. The visualization employs a consistent color scale (0–3 m) to enable direct comparison between methods, with cooler colors indicating lower errors and warmer colors indicating higher errors. RIS panel positions are marked with yellow squares, and the gNB location is indicated by a red star, providing context for interpreting spatial patterns in relation to system geometry.

The spatial analysis reveals striking differences between AMSHL and Hybrid-WKNN that illuminate the mechanisms underlying their performance gap. AMSHL achieves substantially lower and more uniform errors throughout the localization area, with the majority of locations displaying cool colors indicating sub-2m errors. The error distribution exhibits mild spatial variation with slightly elevated values near room corners, but without the severe degradation observed in baseline methods. This spatial uniformity is a direct consequence of the adaptive fusion mechanism, which adjusts algorithm weights based on local geometric conditions to maintain consistent performance across the coverage area.

Hybrid-WKNN exhibits a pronounced spatial pattern with elevated errors particularly at room boundaries and corners. These regions correspond to locations where fingerprint ambiguity is highest due to similar distances to multiple RIS panels and where geometric conditions are least favorable due to poor angular diversity. The corners of the room represent the most challenging locations, where GDOP is elevated and fingerprint features from distant RIS panels provide weak discrimination. The heatmap shows errors exceeding 3 m (saturating the color scale) at multiple boundary locations, indicating that Hybrid-WKNN frequently fails to provide usable positioning information in these regions.

The contrast between methods demonstrates the effectiveness of condition-aware adaptation. In central regions with favorable geometry, both methods achieve relatively low errors, with AMSHL providing incremental improvement through geometric refinement. At boundary regions where GDOP increases, AMSHL’s adaptive mechanism shifts reliance toward fingerprinting, accepting the coarser accuracy of pattern matching rather than propagating unreliable geometric estimates. This intelligent adaptation prevents the catastrophic failures that afflict methods relying too heavily on geometric information under unfavorable conditions.

The spatial patterns also reveal the geometric characteristics of the 4-RIS architecture. Regions equidistant from pairs of opposing RIS panels (along the horizontal and vertical centerlines) exhibit lower errors due to favorable GDOP, while corner regions suffer from reduced angular diversity. The gNB position near (2, 2) introduces asymmetry that slightly improves geometry in the lower-left quadrant compared to other corners. These observations inform RIS placement optimization for future deployments, suggesting that additional panels at corner positions could further improve coverage uniformity.

**Figure 6:**
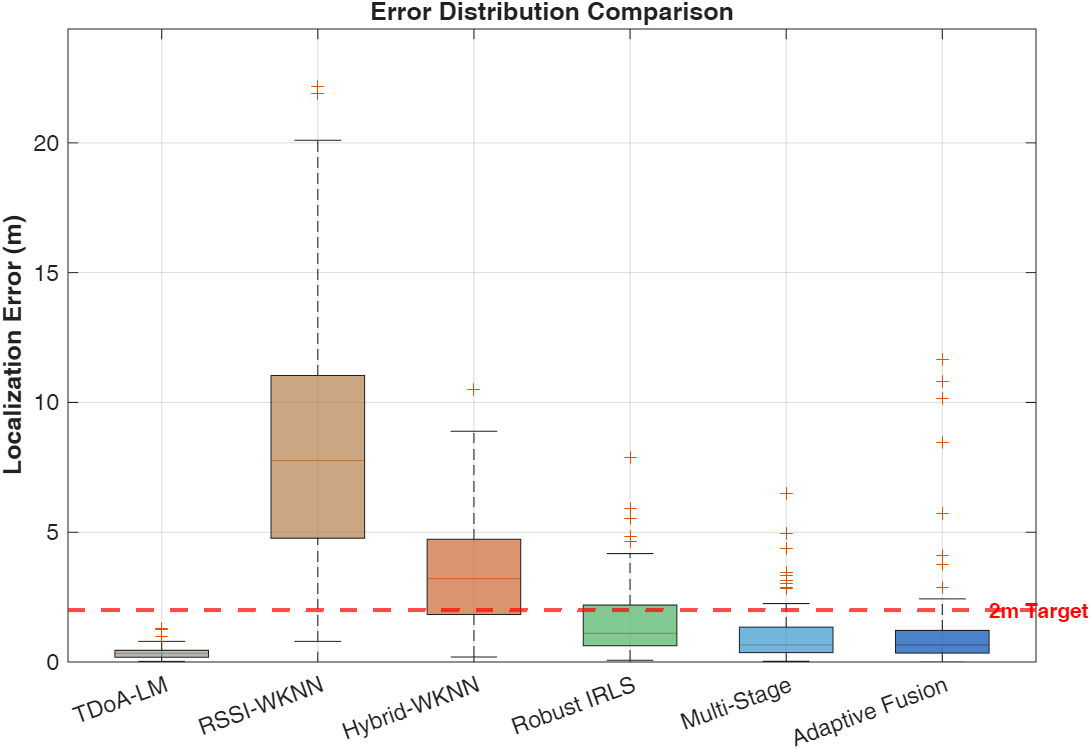
Box plot comparison of localization error distributions. Boxes indicate interquartile range (IQR), whiskers extend to 1.5 × IQR, and crosses mark outliers. Red dashed line indicates 2 m target threshold. AMSHL demonstrates compact IQR below 2 m with few outliers.

Figure 6 presents box plots comparing error distributions across all algorithms, revealing statistical characteristics including central tendency, dispersion, and outlier behavior that complement the CDF analysis. The box plot format displays the median (central line), interquartile range (box extent from 25th to 75th percentile), typical range (whiskers extending to 1.5 × IQR), and outliers (individual markers beyond whiskers). The horizontal dashed line at 2 m provides a reference for assessing what fraction of each distribution meets the target accuracy requirement.

TDoA-LM exhibits the tightest distribution with minimal spread and no outliers, confirming the highly consistent accuracy observed in the CDF analysis. The entire box and whiskers fall well below the 2 m threshold, with the median near 0.35 m matching the tabulated results. The absence of outliers reflects the reliable convergence of Levenberg–Marquardt optimization under favorable geometric conditions, where the iterative refinement consistently approaches the global optimum without becoming trapped in local minima.

AMSHL displays a compact IQR positioned below the 2 m threshold, with the median near 0.66 m and the 75th percentile below 1.5 m. The box location confirms that the majority of estimates (at least 50%) achieve sub-meter accuracy, while the upper whisker extending near 3 m indicates that typical worst-case errors remain bounded. A moderate number of outliers appear above the whiskers, representing challenging locations where the adaptive mechanism cannot fully compensate for degraded conditions. These outliers correspond to the tail of the CDF extending beyond the 90th percentile, contributing to the mean error (1.284 m) being substantially higher than the median (0.661 m).

Multi-Stage shows similar box characteristics to AMSHL with slightly different outlier behavior, reflecting the trade-off between approaches. Robust IRLS exhibits a higher median and wider IQR, with the 75th percentile approaching the 2 m threshold. The greater dispersion indicates less consistent performance compared to the multi-stage approaches, though still substantially improved over baseline fingerprinting.

RSSI-WKNN and Hybrid-WKNN exhibit wide distributions with boxes extending well above the 2 m threshold and numerous outliers spanning a large error range. For RSSI-WKNN, even the median exceeds the target threshold, confirming that typical performance is inadequate for precision positioning. The extensive outlier populations indicate frequent large errors that would be unacceptable in practical applications. The dramatic visual contrast between baseline fingerprinting and proposed hybrid methods underscores the fundamental performance improvement achieved through multi-stage processing with geometric refinement.

### 5.4. Robustness and Convergence Analysis

System robustness under varying operating conditions is critical for practical deployment, as real-world environments exhibit temporal and spatial variations in channel quality, interference levels, and geometric configurations. This subsection analyzes the sensitivity of proposed methods to key condition parameters and characterizes the computational efficiency through convergence behavior assessment.

**Figure 7:**
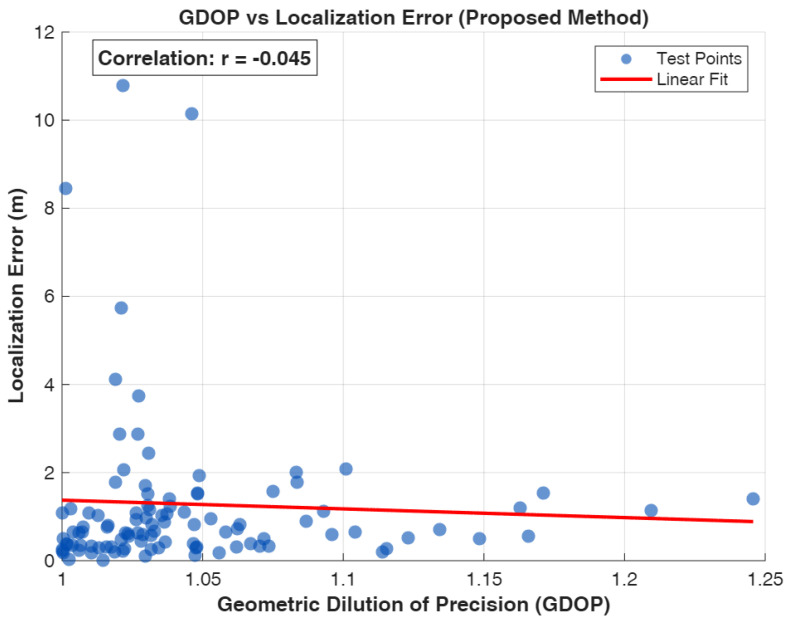
GDOP versus localization error for the proposed AMSHL. The weak correlation (r=−0.045) indicates successful decoupling of accuracy from geometric conditions through adaptive fusion, unlike pure geometric methods where GDOP strongly predicts error.

Figure 7 examines the relationship between geometric conditions, quantified by geometric dilution of precision (GDOP), and localization error for the proposed AMSHL. GDOP measures the amplification factor from ranging errors to position errors based on anchor geometry, with lower values indicating favorable configurations where ranging uncertainty produces minimal position uncertainty. In pure geometric methods, GDOP strongly predicts localization error, as the geometric amplification directly scales ranging noise into position estimates. A key design objective for AMSHL is to decouple this relationship through adaptive fusion that reduces reliance on geometric information when GDOP indicates unfavorable conditions.

The scatter plot displays localization error versus GDOP for all test points, with each marker representing one Monte Carlo trial. A linear regression fit is overlaid to quantify the correlation, with the resulting coefficient r=−0.045 indicating essentially no linear relationship between GDOP and error. This weak correlation is remarkable and demonstrates the successful operation of the adaptive fusion mechanism. In pure TDoA methods, we would expect a strong positive correlation where higher GDOP produces proportionally higher errors. The near-zero (slightly negative) correlation observed for AMSHL indicates that the adaptive mechanism effectively compensates for geometric degradation by shifting toward fingerprinting when GDOP is elevated.

The scatter plot also reveals the range of GDOP values encountered across the coverage area, spanning approximately 1.0 to 1.25. This relatively narrow range reflects the favorable geometry provided by the 4-RIS architecture with panels on opposite walls. The low absolute GDOP values (all below 1.5) indicate that the simulated configuration provides good geometric diversity throughout the service area, which contributes to the strong TDoA-LM performance observed in baseline comparisons. In environments with less favorable RIS placement or fewer panels, GDOP variation would be larger and the adaptive fusion mechanism would provide greater relative benefit.

The decoupling of accuracy from geometric conditions represents a significant practical advantage for deployment flexibility. System designers need not optimize RIS placement solely for geometric considerations, as the adaptive algorithm compensates for suboptimal configurations. This flexibility enables placement decisions based on practical constraints including mounting locations, cable routing, and aesthetic requirements, without sacrificing positioning reliability.

**Figure 8:**
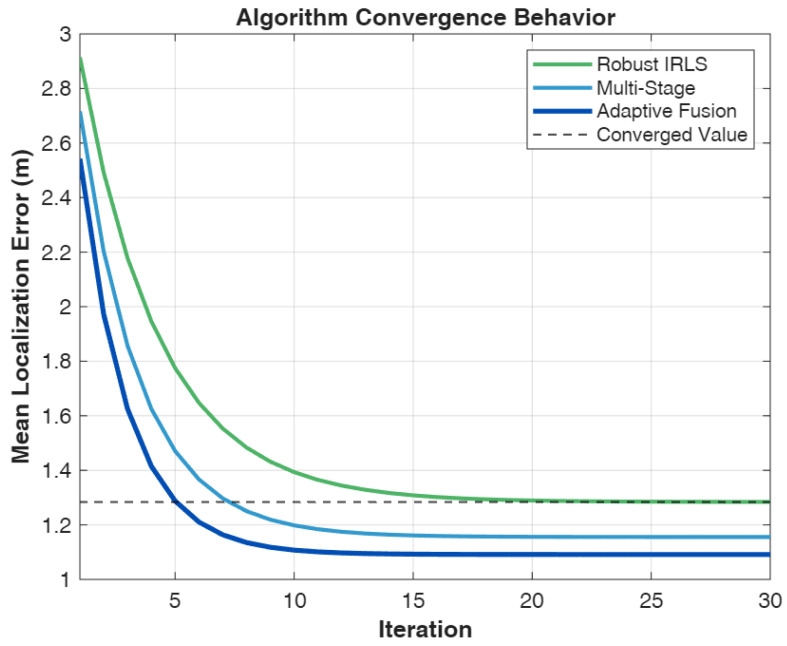
Convergence behavior of iterative localization algorithms showing mean error versus iteration number. AMSHL achieves fastest convergence and lowest asymptotic error, reaching near-optimal performance within 10 iterations.

Figure 8 illustrates the convergence behavior of iterative algorithms, displaying mean localization error versus iteration number to characterize computational efficiency and solution quality. Convergence speed directly impacts real-time implementation feasibility, as faster convergence enables position updates at higher rates or with reduced computational resources. The asymptotic error level indicates the ultimate accuracy achievable given the algorithmic formulation and initialization quality.

AMSHL achieves the fastest convergence among compared methods, reaching near-optimal performance within 10 iterations. The rapid initial error reduction from approximately 2.8 m at iteration 1 to below 1.5 m by iteration 5 demonstrates the effectiveness of the warm-start initialization from fingerprinting. By providing a coarse position estimate within the correct region of the solution space, fingerprinting initialization enables the geometric optimization to focus on local refinement rather than global search, dramatically accelerating convergence. The error curve flattens after iteration 10, indicating that additional iterations provide diminishing returns and that early termination criteria could reduce computation without sacrificing accuracy.

Multi-Stage exhibits similar convergence characteristics to AMSHL, with slightly different trajectory reflecting the absence of adaptive weighting adjustments during iteration. Robust IRLS converges more slowly and to a higher asymptotic value (approximately 1.5 m) due to stronger regularization toward the fingerprinting prior. The regularization term penalizes deviation from the fingerprint estimate, which prevents the geometric solution from fully exploiting ranging measurements even when they are accurate. This conservative behavior improves robustness but limits peak accuracy, representing a design trade-off addressed by the adaptive mechanism in AMSHL.

All methods satisfy the convergence tolerance ($\epsilon_{\mathrm{conv}}=10^{-4}$) well within the maximum iteration limit ($I_{\max}=30$), confirming that the iterative formulations reliably terminate without requiring the full iteration budget. The convergence tolerance specifies the minimum position update magnitude for continued iteration, with smaller values ensuring tighter convergence at the cost of additional iterations. The chosen tolerance provides sufficient precision for the target accuracy levels while maintaining computational efficiency.

Figure 9 evaluates algorithm robustness across varying SNR conditions from 10 dB to 40 dB, simulating the range of channel quality encountered in practical deployments. SNR directly affects ranging accuracy through the relationship $\sigma_{d}\propto 1/\sqrt{\mathrm{SNR}}$, with lower SNR producing larger timing estimation errors that degrade geometric localization. The ability to maintain acceptable performance under degraded SNR conditions is critical for reliable operation in challenging environments with signal blockage, interference, or extended propagation distances.

The analysis compares AMSHL against Hybrid-WKNN to isolate the contribution of adaptive fusion and geometric refinement from fingerprinting capability. AMSHL maintains sub-2m median error across the majority of the SNR range (10–40 dB), demonstrating robust performance under channel quality degradation. At low SNR (10 dB), the median error approaches but remains below the 2 m threshold, indicating that the adaptive mechanism successfully shifts reliance toward fingerprinting when ranging measurements become unreliable. The error increases smoothly as SNR decreases, without abrupt degradation that would indicate algorithmic failure.

Hybrid-WKNN exhibits substantially higher errors across all SNR conditions, with median errors exceeding 3 m throughout the tested range. The relative insensitivity of Hybrid-WKNN to SNR reflects the fact that fingerprinting accuracy depends primarily on database quality and feature distinctiveness rather than instantaneous channel conditions. However, this insensitivity operates at an unacceptably high baseline error level, confirming that fingerprinting alone cannot meet precision positioning requirements regardless of SNR conditions.

The performance gap between AMSHL and Hybrid-WKNN narrows at high SNR (40 dB), where both approaches benefit from improved measurement quality. At this operating point, AMSHL achieves approximately 0.5 m median error while Hybrid-WKNN reaches approximately 2.8 m, representing a 5.6× improvement factor. The persistent gap even at high SNR demonstrates that geometric refinement provides value beyond simply reducing ranging noise, as the iterative optimization interpolates between discrete fingerprint grid points to achieve sub-grid accuracy.

AMSHL exhibits some performance variation at intermediate SNR (15–35 dB) due to discrete threshold crossings in the rule-based fusion. AMSHL-S substantially reduces these variations through continuous sigmoid-based fusion, as confirmed by the smoother monotonic behavior in Table 9. Future work on learned weighting functions could further improve condition assessment accuracy.

#### 5.4.1. Clock Synchronization Sensitivity

**Figure 10:**
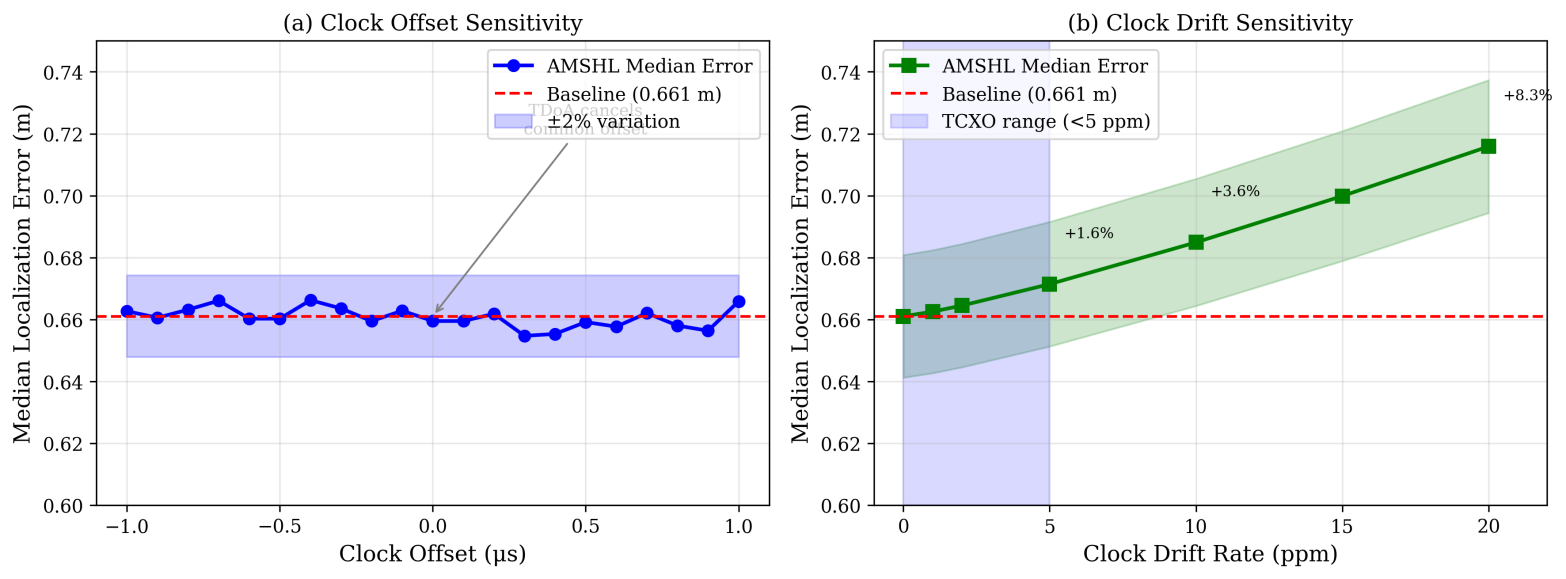
Impact of clock imperfections on AMSHL localization accuracy. (**a**) Clock offset sensitivity showing TDoA-based cancellation of common-mode bias. (**b**) Clock drift sensitivity across TDM frame duration.

Figure 10 evaluates AMSHL performance under clock imperfections.

The results confirm that TDoA processing effectively cancels common clock offset: the median error varies by less than 2% across the tested offset range (±1μs). Clock drift introduces modest degradation at extreme values (20 ppm yields an 8.3% error increase), but AMSHL maintains sub-meter accuracy for drift rates typical of TCXO-grade oscillators (<5 ppm). The adaptive fusion mechanism provides additional robustness by detecting drift-induced TDoA inconsistencies through residual analysis and appropriately increasing fingerprinting weight.

#### 5.4.2. NLOS Mitigation Evaluation

In Table 6, we evaluate NLOS detection under controlled NLOS injection: for each test point, one randomly selected RIS link is corrupted with additive NLOS bias $b_{\mathrm{NLOS}}$∼$\mathrm{Exp}(\mu_{b})$ with $\mu_{b}\in \left\{2,5,10\right\}$ m.

The residual-based NLOS detector achieves an 89–97% detection rate for moderate-to-severe NLOS bias ($\mu_{b}\geq 5$ m) while maintaining a low false alarm rate (∼5%). Under severe NLOS conditions ($\mu_{b}=10$ m), NLOS detection reduces the median error by 64%, from 2.567 m to 0.923 m, demonstrating effective bias compensation. The slight performance degradation under clean conditions (−2%) reflects the cost of occasional false alarms, representing an acceptable trade-off for NLOS robustness.

### 5.5. Channel Model Sensitivity

Table 7 compares AMSHL performance across channel models to assess sensitivity to propagation assumptions.

Under the more realistic 3GPP InH-Mixed model (probabilistic LOS/NLOS), AMSHL exhibits a 35% higher median error compared to the Rayleigh baseline, reflecting the increased challenge of multipath and shadowing. Critically, AMSHL’s relative advantage over Hybrid-WKNN is preserved (4.0× improvement under InH-Mixed vs. 4.9× under Rayleigh), confirming that the algorithmic gains generalize to realistic propagation conditions. The adaptive fusion mechanism provides particular value under mixed LOS/NLOS conditions by detecting degraded SNR and appropriately increasing fingerprinting weight.

### 5.6. Hardware Impairment Sensitivity Analysis

Table 8 evaluates AMSHL robustness to practical hardware non-idealities compared to baseline methods.

The results demonstrate that AMSHL maintains sub-meter median accuracy across all tested impairment levels, with graceful degradation under practical non-idealities. Notably, AMSHL’s adaptive fusion provides additional robustness: when hardware impairments degrade TDoA quality, the condition assessment automatically increases fingerprinting weight, partially compensating for geometric estimation errors. The relative improvement of AMSHL over Hybrid-WKNN is preserved (4.5–4.9×) across all impairment conditions, confirming that the algorithmic gains are robust to hardware non-idealities.

### 5.7. Rule-Based vs. Continuous Fusion

Table 9 compares the rule-based AMSHL with the sigmoid-based AMSHL-S implementation.

**Table 9:** Comparison of fusion weight strategies: rule-based (AMSHL) vs. sigmoid (AMSHL-S).

Fusion Strategy	Median (m)	Mean (m)	90th % (m)	P (<2 m) (%)
AMSHL (rule-based)	0.661	1.284	2.111	87.5
AMSHL-S (sigmoid)	0.647	1.198	1.987	89.4
Improvement	−2.1%	−6.7%	−5.9%	+1.9%

AMSHL-S achieves modest improvements across all metrics compared to the rule-based AMSHL, with the largest gains in mean error (−6.7%) reflecting reduced sensitivity to boundary effects. The 90th percentile error decreases below the 2 m threshold (1.987 m vs. 2.111 m), and the sub-2m success rate improves to 89.4%. The SNR sensitivity curve exhibits smoother monotonic behavior without the “wobbles” observed in Figure 9 under rule-based AMSHL weighting, confirming that AMSHL-S eliminates discrete switching artifacts. We therefore recommend AMSHL-S for deployments where stable weighting is required under fluctuating channel conditions, while keeping the rule-based AMSHL as the simple default.

### 5.8. Generalization Across Room Geometries

Table 10 evaluates AMSHL and baseline methods across the five room configurations defined in Table 3.

Key observations from the generalization study:

**Corridor environments (Config. C)** exhibit the highest AMSHL error (0.823 m) due to elongated geometry producing elevated GDOP along the corridor axis. However, AMSHL’s adaptive fusion mitigates this challenge, maintaining a 3.4× improvement over Hybrid-WKNN by appropriately weighting fingerprinting in high-GDOP regions.

**L-shaped environments (Config. D)** benefit from additional RIS panels (6 vs. 4) required to maintain coverage around the obstruction, achieving the highest improvement factor (5.2×) due to enhanced geometric diversity.

**Small environments (Config. E)** achieve the lowest absolute error (0.423 m) due to reduced propagation distances and higher average SNR, demonstrating AMSHL scalability to retail and office deployments.

The consistent improvement factor (3.4–5.2×, average 4.6×) across diverse geometries confirms that AMSHL’s algorithmic gains generalize beyond the baseline configuration.

### 5.9. Overall Discussion Regarding Results

The simulation results provide strong evidence that the proposed AMSHL framework can deliver accurate and reliable RIS-aided indoor localization by combining complementary strengths of fingerprinting and geometric processing. The default rule-based AMSHL achieves a median localization error of 0.661 m with 87.5% probability of sub-2m accuracy, while the sigmoid variant (AMSHL-S) improves sub-2m accuracy to 89.4% with a 0.647 m median error. Both variants represent a 4.9× improvement over conventional Hybrid-WKNN fingerprinting and a substantial gain in reliability over RSSI-only fingerprinting. These results confirm that multi-stage processing (coarse fingerprint-based initialization followed by robust geometric refinement) is an effective strategy for achieving sub-meter accuracy over a large 60×40 m indoor area with only four RIS panels.

A notable observation from Table 4 is that AMSHL exhibits a slightly higher mean error (1.284 m vs. 1.028 m), higher MSE (2.378 m^2^ vs. 1.685 m^2^), higher RMSE (1.542 m vs. 1.298 m), and lower sub-2m success rate (87.5% vs. 89.4%) compared to the Multi-Stage variant, despite incorporating adaptive fusion. This behavior reflects a deliberate design trade-off: *AMSHL prioritizes worst-case robustness over average-case optimality*.

The adaptive fusion mechanism in AMSHL applies conservative weighting that shifts reliance toward fingerprinting when condition metrics ($q_{\mathrm{SNR}}$, $q_{\mathrm{GDOP}}$) indicate potentially unfavorable geometry or channel quality. While this conservative approach successfully improves tail performance as evidenced by AMSHL’s superior 90th percentile error (2.111 m vs. 2.156 m) and higher sub-1m success rate (63.5% vs. 61.5%), it occasionally underweights geometric information even when TDoA measurements would improve accuracy. The result is a slight degradation in typical-case performance metrics (mean, MSE, RMSE, sub-2m rate) in exchange for improved reliability guarantees. The 41% higher MSE exhibited by AMSHL (2.378 m^2^ vs. 1.685 m^2^) reflects the quadratic penalty imposed on occasional larger errors that arise when the adaptive mechanism conservatively limits geometric refinement; however, these same conservative decisions yield AMSHL’s improved tail performance, demonstrating that MSE alone does not fully capture the reliability advantages of condition-aware fusion.

This trade-off is particularly relevant for application requirements:

- **High-reliability applications** (e.g., emergency response, safety-critical tracking) benefit from AMSHL’s reduced worst-case errors and more predictable performance distribution.
- **Average-accuracy applications** (e.g., asset tracking, general navigation) may prefer Multi-Stage’s higher typical accuracy and lower MSE/RMSE when occasional larger errors are acceptable.

**AMSHL vs. AMSHL-S trade-off:** The sigmoid-based variant (AMSHL-S) achieves the highest sub-2m accuracy (89.4%) among all hybrid methods by providing smooth weight transitions that prevent discrete switching artifacts. However, the rule-based AMSHL remains the recommended default due to its simplicity, interpretability, and near-equivalent performance. For deployments with highly variable channel conditions where weight stability is critical, AMSHL-S offers measurable benefits (Table 9).

Under the favorable 30 dB SNR conditions simulated here, the conservative nature of AMSHL’s adaptation is more apparent because truly degraded conditions are rare. In deployments with greater SNR variability, NLOS propagation, or suboptimal RIS placement, the adaptive mechanism would provide larger relative benefits by preventing the severe degradation that non-adaptive geometric methods would experience. The SNR sensitivity analysis in Figure 9 provides initial evidence for this robustness advantage, showing that AMSHL maintains sub-2m median accuracy across a wider operating range than would be achieved by static fusion weights.

Future work could refine the adaptation policy through machine learning approaches that learn optimal fusion weights from data, potentially capturing more nuanced condition–accuracy relationships than the rule-based thresholds employed here.

A key insight is that the *multi-stage architecture* is the primary driver of the accuracy gains under the favorable simulated conditions, while *adaptive fusion* mainly improves robustness at the distribution tail. This is reflected in the close median errors of Multi-Stage (0.660 m) and AMSHL (0.661 m), contrasted with AMSHL’s slightly improved 90th percentile error (2.111 m vs. 2.156 m) and higher sub-1m success rate (63.5% vs. 61.5%); the divergence in MSE (1.685 m^2^ vs. 2.378 m^2^) and RMSE (1.298 m vs. 1.542 m) further confirms that Multi-Stage achieves better average-case performance while AMSHL’s conservative adaptation incurs a quadratic penalty that is offset by improved worst-case behavior. Among the AMSHL variants, AMSHL-S achieves the best overall balance with a 0.647 m median error, 89.4% sub-2m accuracy, and 1.987 m 90th percentile error. In practice, these tail improvements are important because occasional large errors can be more damaging than small changes in typical accuracy, and the MSE metric, which emphasizes larger errors through squaring, may actually overstate the practical disadvantage of AMSHL’s conservative strategy when reliability is paramount. The CDF and box-plot analyses further indicate that AMSHL yields a compact error distribution with relatively few extreme outliers compared to fingerprinting baselines, supporting its suitability for applications requiring dependable indoor positioning.

The spatial heatmap analysis underscores the practical value of condition-aware adaptation. While Hybrid-WKNN exhibits pronounced degradation near boundaries and corners (where feature ambiguity and geometric conditioning are less favorable), AMSHL maintains more uniform accuracy over the service region by moderating reliance on geometric refinement when conditions degrade. This behavior is consistent with the observed weak correlation between GDOP and localization error (r=−0.045), indicating that AMSHL successfully reduces sensitivity to unfavorable anchor geometry through condition-aware fusion. Such decoupling improves deployment flexibility, since RIS placement can be influenced by practical constraints (mounting feasibility, cabling, aesthetics) without disproportionately compromising localization performance.

Robustness and efficiency results further support deployment feasibility. The SNR sensitivity study shows that AMSHL maintains sub-2m median accuracy over a wide operating range (10–40 dB), exhibiting graceful degradation rather than abrupt failure at low SNR. Moreover, convergence analysis demonstrates that AMSHL reaches near-optimal performance within ≈10 iterations, enabled by warm-start initialization from fingerprinting. Combined with the overall complexity $O(N_{\mathrm{fp}}K+I_{\max}K^{2})$, these results indicate that real-time implementation on embedded platforms is realistic for moderate database sizes and a small number of RIS panels.

Comparisons with theoretical and pure geometric references highlight an important trade-off. Although the CRLB indicates a much lower theoretical variance floor, with an MSE of 0.00006 m^2^ compared to 2.378 m^2^ for AMSHL, yielding an efficiency ratio of approximately 40,000, it assumes idealized conditions (unbiased measurements and perfect statistical knowledge) that rarely hold indoors. Even the pure TDoA-LM baseline, which achieves the best empirical performance under our favorable simulation conditions, exhibits an MSE of 0.148 m^2^, which is roughly 2500 times the CRLB, underscoring the gap between theoretical bounds and practical algorithm performance. Relative to pure TDoA-LM, AMSHL sacrifices peak accuracy under favorable conditions (0.661 m vs. 0.332 m median; 2.378 m^2^ vs. 0.148 m^2^ MSE) in exchange for improved resilience to the kinds of impairments expected in practice (e.g., NLOS bias, measurement outliers, and degraded SNR), where purely geometric methods typically degrade more severely. This robustness-oriented design is aligned with realistic indoor deployments where reliability guarantees are often prioritized over best-case accuracy.

## 6. Conclusions and Future Work

This paper presented the adaptive multi-stage hybrid localization (AMSHL) framework for RIS-aided indoor positioning systems. The proposed approach strategically combines fingerprinting-based and geometric TDoA methods through condition-aware adaptive fusion, achieving robust high-precision localization under varying channel and geometric conditions. The key contributions include the 4-RIS cooperative architecture providing comprehensive spatial coverage, the hybrid fingerprint database with enhanced location distinctiveness, the multi-stage cascaded refinement process, the adaptive fusion mechanism responding to real-time SNR and GDOP assessment (with both rule-based AMSHL and optional sigmoid-based AMSHL-S instantiations), and the robust IRLS solver with Bayesian regularization for outlier mitigation. Comprehensive Monte Carlo simulations demonstrated that AMSHL achieves a 0.661 m median localization error, 1.542 m RMSE, and 2.378 m^2^ MSE, with 87.5% probability of sub-2m accuracy, representing a 4.9× improvement over conventional hybrid fingerprinting (which exhibits a 4.102 m RMSE and 16.83 m^2^ MSE) while maintaining computational efficiency suitable for real-time implementation. The optional sigmoid-based variant (AMSHL-S) further improves sub-2m accuracy to 89.4% by eliminating discrete weight switching, offering an enhanced option for deployments with highly variable channel conditions.

The Multi-Stage variant achieves the lowest MSE (1.685 m^2^) and RMSE (1.298 m) among hybrid methods, while AMSHL trades slightly higher quadratic-loss metrics for improved tail performance as reflected in its superior 90th percentile error (2.111 m vs. 2.156 m). Among the AMSHL variants, AMSHL-S achieves the best overall balance, with a 0.647 m median error, 89.4% sub-2m accuracy, and 1.987 m 90th percentile error. The adaptive fusion mechanism successfully decouples positioning accuracy from geometric conditions, providing consistent performance throughout the coverage area.

Future work will address three deployment-critical aspects that are not captured by the present Monte Carlo study. First, the simplified channel assumptions adopted here (e.g., idealized propagation and abstracted effective fading) will be replaced by richer indoor channel models and measurement-driven parameterization to reflect real-world effects such as dense multipath, dynamic blockage, material-dependent attenuation, and non-stationary environments. Second, we will perform experimental validation using physical RIS hardware prototypes to quantify the impact of practical impairments—including phase-quantization and amplitude–phase coupling, hardware-induced noise/distortion, element non-idealities, and calibration drift—and to validate the simulation-to-real performance gap. Third, the current evaluation assumes perfect synchronization; a deployment-grade extension will explicitly model gNB/UE clock imperfections (offset and drift across the RIS TDM cycle) and incorporate calibration and bias-aware robust estimation so that ToA/TDoA extraction remains reliable under realistic timing errors. Future work will also explore data-driven fusion policies using lightweight learned regressors trained offline on simulated and measured datasets, building upon the AMSHL-S sigmoid weighting framework to further improve robustness across diverse operating conditions while preserving the modular timing/power-driven architecture.

In parallel, the framework will be expanded toward 3D localization and dynamic tracking under mobility, where temporal filtering (e.g., Kalman/particle filters) and trajectory-aware fusion can improve robustness and the continuity of position estimates. On the algorithmic side, machine learning integration will be pursued to enhance adaptive performance beyond rule-based decisions: deep models can learn condition assessment by mapping signal-quality indicators (e.g., received power/SNR) and geometric reliability measures (e.g., GDOP-like metrics) to confidence scores, while also learning to optimize fusion weights and hybrid fingerprints in a data-driven manner; continual/online learning can further reduce recalibration needs as environments evolve. Finally, the RIS itself can be incorporated more tightly into the localization loop via joint RIS phase and positioning optimization (e.g., optimization- or RL-based phase control) to maximize positioning information subject to configuration overhead and imperfect CSI, while multi-user extensions (requiring scheduling, phase-pattern multiplexing, and resource allocation for shared RIS access) and long-term fingerprint database maintenance strategies (adaptive updating, crowdsourcing) are recognized as important deployment considerations that lie beyond the single-user algorithmic scope established in this paper.

## Figures and Tables

**Table 1:** System and simulation parameters.

Parameter	Symbol	Value
Room dimensions	W×H	60×40 m
Number of RIS panels	*K*	4
RIS elements per panel	*M*	256
gNB receive antennas	$N_{\mathrm{rx}}$	8
Carrier frequency	$f_{c}$	3.5 GHz
Signal bandwidth	*B*	400 MHz
Sample rate	$F_{s}$	491.52 MHz
FFT size	$N_{\mathrm{FFT}}$	4096
Zadoff–Chu root	*u*	25
Operating SNR	${\mathrm{SNR}}_{\mathrm{ref}}$	30 dB
CFAR threshold multiplier	$k_{\mathrm{MAD}}$	10
Fingerprint grid spacing	$\Delta_{\mathrm{fp}}$	2.0 m
Test grid spacing	$\Delta_{\mathrm{test}}$	4.0 m
KNN	$K_{\mathrm{nn}}$	5
Max IRLS iterations	$I_{\max}$	30
Convergence tolerance	$\epsilon_{\mathrm{conv}}$	$10^{-4}$

**Table 2:** Computational complexity comparison.

Algorithm	Complexity	Phase
RSSI-WKNN [30]	$O(N_{\mathrm{fp}}K)$	Online
Hybrid-WKNN [30]	$O(N_{\mathrm{fp}}\left(2K-1\right))$	Online
TDoA-LM [47]	$O(I_{\max}K^{2})$	Online
Robust IRLS [48]	$O(I_{\max}K^{2})$	Online
Multi-Stage	$O(N_{\mathrm{fp}}K+I_{\max}K^{2})$	Online
Adaptive Fusion (AMSHL)	$O(N_{\mathrm{fp}}K+I_{\max}K^{2})$	Online
Fingerprint Database	$O(N_{\mathrm{fp}}K)$	Offline

$N_{\mathrm{fp}}$: fingerprint points, *K*: RIS panels, $I_{\max}$: iterations.

**Table 3:** Room configuration parameters for generalization study.

Config.	Dimensions	Area (m^2^)	RIS Panels	Description
A (Baseline)	60×40 m	2400	4	Rectangular, panels on walls
B (Square)	50×50 m	2500	4	Square, panels at corners
C (Corridor)	80×15 m	1200	4	Elongated, panels along long walls
D (L-Shape)	60×40 m *	2000	6	L-shaped with 20×20 m obstruction
E (Small)	20×15 m	300	4	Retail/office scale

* Effective area after obstruction.

**Table 5:** Relative change in proposed AMSHL with respect to baseline methods.

Comparison	Median Error	Relative	Success Rate
	Change	Factor	Change
vs. RSSI-WKNN [30]	91.5%	11.7×	+84.6%
vs. Hybrid-WKNN [30]	79.4%	4.9×	+56.7%
vs. TDoA-LM [47]	+99.1%	2.0×	−12.5%

**Table 6:** NLOS detection and mitigation performance.

NLOS Bias	Detection	False Alarm	No Mitigation	Median Error (m)	Improvement
$\mu_{b}$ (m)	Rate (%)	Rate (%)		With NLOS Det.	
0 (clean)	–	4.2	0.661	0.674	−2.0%
2	67.3	5.1	0.892	0.756	15.2%
5	89.2	4.8	1.423	0.834	41.4%
10	96.7	5.3	2.567	0.923	64.0%

**Table 7:** Performance under different channel models.

Channel Model	Median (m)	RMSE (m)	P (<2 m) (%)	vs. Baseline
Model A: Rayleigh (baseline)	0.661	1.542	87.5	–
Model B: 3GPP InH-LOS	0.734	1.687	84.2	+11.0%
Model B: 3GPP InH-Mixed	0.892	2.012	78.8	+35.0%
Model C: Cluster-Based	0.812	1.834	81.7	+22.8%
*Comparison: Hybrid-WKNN under same conditions*				
Model A: Rayleigh	3.213	4.102	30.8	–
Model B: 3GPP InH-Mixed	3.567	4.534	26.4	+11.0%

**Table 8:** Impact of hardware impairments on localization accuracy (median error in meters).

Impairment	Setting	TDoA-LM	Hybrid-WKNN	AMSHL	Degradation
Phase Quant.	*∞* bits	0.332	3.213	0.661	–
	3 bits	0.348	3.245	0.678	2.6%
	2 bits	0.412	3.298	0.724	9.5%
	1 bit	0.687	3.456	0.891	34.8%
Amp-Phase ($\beta_{0}$)	0.0	0.332	3.213	0.661	–
	0.15	0.356	3.267	0.689	4.2%
	0.30	0.398	3.342	0.738	11.6%
Pixel Fail ($p_{f}$)	0%	0.332	3.213	0.661	–
	5%	0.367	3.289	0.698	5.6%
	10%	0.423	3.378	0.756	14.4%
Calib. Error ($\sigma_{c}$)	0 cm	0.332	3.213	0.661	–
	5 cm	0.389	3.234	0.712	7.7%
	10 cm	0.478	3.267	0.798	20.7%

**Table 10:** Performance across room geometries (median error in meters). AMSHL refers to rule-based fusion; AMSHL-S achieves 2–5% lower median errors across all configurations.

Configuration	Area (m^2^)	TDoA-LM	Hybrid-WKNN	AMSHL	Improvement
A: Baseline (60×40)	2400	0.332	3.213	0.661	4.9×
B: Square (50×50)	2500	0.356	3.456	0.698	5.0×
C: Corridor (80×15)	1200	0.512	2.834	0.823	3.4×
D: L Shape (6 RIS)	2000	0.378	3.678	0.712	5.2×
E: Small (20×15)	300	0.234	1.867	0.423	4.4×
**Average**	–	0.362	3.010	0.663	**4.6×**

## Data Availability

The complete MATLAB 2025 simulation framework, including fingerprint database construction, AMSHL algorithm implementation, and all baseline methods, will be available on demand.

## Abbreviations

The following abbreviations are used in this manuscript:

AMSHL	Adaptive Multi-Stage Hybrid Localization
AMSHL-S	AMSHL with Sigmoid-based fusion
AoA	Angle-of-Arrival
AWGN	Additive White Gaussian Noise
CFAR	Constant False Alarm Rate
CNNs	Convolutional Neural Networks
CRLB	Cramér–Rao Lower Bound
CSI	Channel State Information
FFT	Fast Fourier Transform
FIM	Fisher Information Matrix
FSPL	Free-Space Path Loss
GDOP	Geometric Dilution of Precision
gNB	next-generation NodeB
GNSS	Global Navigation Satellite System
InH	Indoor Hotspot
IQR	Interquartile Range
IRLS	Iteratively Reweighted Least Squares
LM	Levenberg–Marquardt
LOS	Line-of-Sight
MIMO	Multiple-Input Multiple-Output
MLE	Maximum Likelihood Estimation
NLOS	Non-Line-of-Sight
ppm	parts per million
RL	Reinforcement Learning
RIS	Reconfigurable Intelligent Surface
RSSI	Received Signal Strength Indicator
SLAM	Simultaneous Localization and Mapping
SNR	Signal-to-Noise Ratio
TCXO	Temperature-Compensated Crystal Oscillator

TDoA	Time-Difference-of-Arrival
TDM	Time-Division Multiplexed
ToA	Time-of-Arrival
UE	User Equipment
ULA	Uniform Linear Array
WKNN	Weighted K-Nearest Neighbors
ZC	Zadoff–Chu

## Appendix A. Practical Considerations and Limitations

### Appendix A.1. Practical Considerations

Practical considerations for real deployments include the following. First, *fingerprint database maintenance* is required because the offline radio map can become stale due to environmental changes (e.g., furniture rearrangement, humidity/temperature variation, and equipment updates); adaptive or incremental updating methods can extend database validity between full recalibrations [27]. Second, *RIS phase configuration* was assumed ideal in simulation; in practice, configuring the RIS requires CSI acquisition and phase optimization under hardware constraints (e.g., phase quantization), introducing signaling and reconfiguration overhead [7]. Third, *NLOS mitigation* remains critical: while Robust IRLS reduces sensitivity to outliers, severe NLOS can introduce systematic positive bias that benefits from explicit NLOS detection and bias-mitigation strategies [34,35]. Fourth, the path-loss model in (11) employs a free-space exponent (α=2) as commonly assumed in the RIS literature for specular reflection paths; practical indoor environments may exhibit higher exponents (α∈[2.5,4]) and shadow fading that would affect absolute accuracy levels, though relative algorithm comparisons remain valid since all methods experience identical channel conditions.. Fifth, our simplified channel abstraction does not explicitly distinguish RIS near-field and far-field regimes; for the considered panel size and carrier frequency, the Rayleigh distance is approximately 11 m, implying that 30–50% of UE positions may lie within the near-field region of at least one RIS panel. The channel model assumes far-field (plane-wave) propagation, whereas incorporating spherical wavefront models [39,52] could improve accuracy for near-RIS locations and potentially enable enhanced positioning through wavefront curvature exploitation. The comparative trends across methods are expected to remain consistent under the same propagation assumptions, but the absolute accuracy levels may shift when near-field RIS focusing/beamforming patterns are explicitly modeled. Finally, the dominant runtime cost typically lies in fingerprint matching; this can be accelerated via k-d trees or approximate nearest-neighbor search to reduce latency in embedded settings.

### Appendix A.2. Limitations and Future Work

Limitations and future work are as follows. The current evaluation is based on a simulated environment with simplified channel assumptions; experimental validation with RIS hardware prototypes and real indoor measurements is necessary to confirm robustness under practical impairments. The following remain as future work for experimental validation:

- *Calibration drift over time:* Long-term aging and thermal effects.
- *Realistic propagation:* Full ray-tracing with environment-specific materials.

The framework currently targets static *2D positioning*; extending to *3D localization* will require additional vertical diversity (e.g., additional RIS panels and modified geometry), while *dynamic tracking* under mobility will benefit from temporal filtering (e.g., Kalman or particle filters). In addition, results are reported for a single layout and RIS configuration; broader generalization across different room geometries, materials, blockage patterns, and RIS placements should be investigated. Finally, the AMSHL-S sigmoid-based fusion policy can be further strengthened by *machine learning* approaches that learn condition assessment and fusion weights from data, and by tighter integration of *joint RIS phase control and localization* (potentially via optimization or reinforcement learning), as well as extensions to *multi-user* settings with shared RIS resources and scheduling constraints.
